# Tumor-infiltrating exhausted CD8^+^ T cells dictate reduced survival in premenopausal estrogen receptor–positive breast cancer

**DOI:** 10.1172/jci.insight.153963

**Published:** 2022-02-08

**Authors:** Colt A. Egelston, Weihua Guo, Jiayi Tan, Christian Avalos, Diana L. Simons, Min Hui Lim, Yinghui J. Huang, Michael S. Nelson, Arnab Chowdhury, Daniel B. Schmolze, John H. Yim, Laura Kruper, Laleh Melstrom, Kim Margolin, Joanne E. Mortimer, Yuan Yuan, James R. Waisman, Peter P. Lee

**Affiliations:** 1 Department of Immuno-Oncology, Beckman Research Institute;; 2 Light Microscopy Digital Imaging Core, Beckman Research Institute;; 3 Division of Biostatistics, Department of Computational and Quantitative Medicine, Beckman Research Institute; and; 4 Department of Pathology,; 5 Department of Surgery, and; 6 Department of Medical Oncology & Therapeutics Research, City of Hope, Duarte, California, USA.

**Keywords:** Immunology, Oncology, Breast cancer, T cells

## Abstract

CD8^+^ tumor-infiltrating lymphocytes (TILs) are associated with improved survival in triple-negative breast cancer (TNBC) yet have no association with survival in estrogen receptor–positive (ER^+^) BC. The basis for these contrasting findings remains elusive. We identified subsets of BC tumors infiltrated by CD8^+^ T cells with characteristic features of exhausted T cells (T_EX_). Tumors with abundant CD8^+^ T_EX_ exhibited a distinct tumor microenvironment marked by amplified interferon-γ signaling–related pathways and higher programmed death ligand 1 expression. Paradoxically, higher levels of tumor-infiltrating CD8^+^ T_EX_ associated with decreased overall survival of patients with ER^+^ BC but not patients with TNBC. Moreover, high tumor expression of a CD8^+^ T_EX_ signature identified dramatically reduced survival in premenopausal, but not postmenopausal, patients with ER^+^ BC. Finally, we demonstrated the value of a tumor T_EX_ signature score in identifying high-risk premenopausal ER^+^ BC patients among those with intermediate Oncotype DX Breast Recurrence Scores. Our data highlight the complex relationship between CD8^+^ TILs, interferon-γ signaling, and ER status in BC patient survival. This work identifies tumor-infiltrating CD8^+^ T_EX_ as a key feature of reduced survival outcomes in premenopausal patients with early-stage ER^+^ BC.

## Graphical abstract

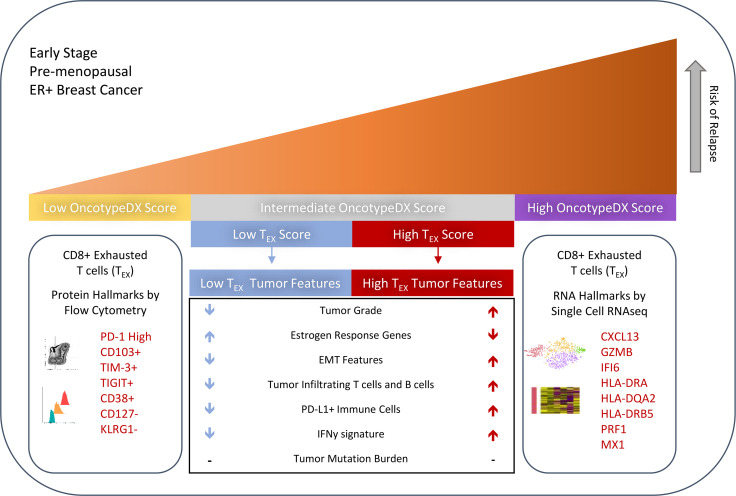

## Introduction

In most cancer types, presence of tumor-infiltrating lymphocytes (TILs) denotes reduced risk for relapse and increased overall survival (1). The prognostic impact of CD8^+^ TILs appears to be subtype specific in breast cancer (BC) (2, 3). CD8^+^ tumor-infiltrating T cells positively associate with survival in triple-negative BC (TNBC) and human epidermal growth factor receptor 2 (HER2/neu) overexpressed (HER2^+^) BC, but not in estrogen receptor–positive (ER^+^) BC (4–6). This paradox is further complicated by differences in kinetics of disease progression among BC subtypes. As compared with patients with TNBC, patients with ER^+^ BC rarely have early relapse events but have a higher overall relapse rate more than 5 years after diagnosis (7). A better understanding of the relationship between TILs and patient outcomes is needed to guide therapeutic strategies for patients with ER^+^ BC, who compose approximately 70% of all patients with BC (8).

Progress in dissecting the complexity of CD8^+^ TIL heterogeneity has shed light on the role of specific T cell subsets in antitumor immunity. We and others have shown that primary tumor-infiltrating resident memory T cells, a subset of CD8^+^ T cells that localize within peripheral tissue without recirculation, positively associate with increased survival in patients with TNBC (9, 10). Tumor infiltration of granzyme B^+^CD8^+^ TILs and an interferon-γ (IFN-γ) signature also denote favorable outcomes in patients with TNBC (11). However, a detailed understanding of the relationship between CD8^+^ TIL subsets and ER^+^ BC patient survival characteristics is still lacking.

More recently, the relationship between antitumor immunity and a CD8^+^ T cell subset termed exhausted T cells (T_EX_) has become better understood. CD8^+^ T_EX_ are generally described as cells with reduced production capacity of cytokines IFN-γ, TNF-α, and IL-2 (12). CD8^+^ T_EX_ also express elevated levels of immune checkpoint molecules, including programmed death 1 (PD-1), TIM-3 (encoding T cell Ig and mucin domain-containing protein 3), and cytotoxic T lymphocyte–associated protein 4 (CTLA-4) (13, 14). PD-1 “high” expression by T cells has long been regarded as a marker of T cell dysfunction and more recently has been recognized as a surrogate marker for tumor specificity (15–17). We previously showed that PD-1^+^ functional cells predominate the CD8^+^ tumor infiltrate in most primary breast tumors (18). In addition to PD-1, expression of the ectoenzyme CD39 has been reported to reliably mark CD8^+^ T_EX_ in both cancer and infectious disease settings (19, 20). It has been further demonstrated that PD-1^+^CD39^+^CD8^+^ T_EX_ are tumor specific, are associated with good prognosis in head and neck cancer patients, and predict response to checkpoint blockade in lung cancer patients (21, 22). Together, these reports have defined CD8^+^ T_EX_ as important mediators of antitumor immunity and biomarkers of clinical significance.

Here, we show that tumor-infiltrating CD8^+^ T_EX_ are detectable in a subset of both ER^+^ and TNBC tumors. We show that BC patient tumors enriched with CD8^+^ T_EX_ have distinct tumor microenvironment (TME) immune composition and increased IFN-γ–related activity. We demonstrate a CD8^+^ T_EX_ signature that delineates patients with ER^+^ BC with marked differences in overall survival. Intriguingly, we find that high CD8^+^ T_EX_ tumor infiltration identifies a subset of premenopausal patients with ER^+^ BC with decreased overall survival and relapse-free survival. Finally, we demonstrate the utility of complementing Oncotype DX scoring with T_EX_ signature scoring to identify high-risk premenopausal patients with ER^+^ BC. Together, these findings unravel what we believe are previously unidentified relationships between CD8^+^ T cell tumor infiltration and patient prognosis and highlight CD8^+^ T_EX_ as a critical feature of ER^+^ BC patient outcomes.

## Results

### Exhausted CD8^+^ T cells are enriched in subsets of BC patient tumors.

**Figure 1:**
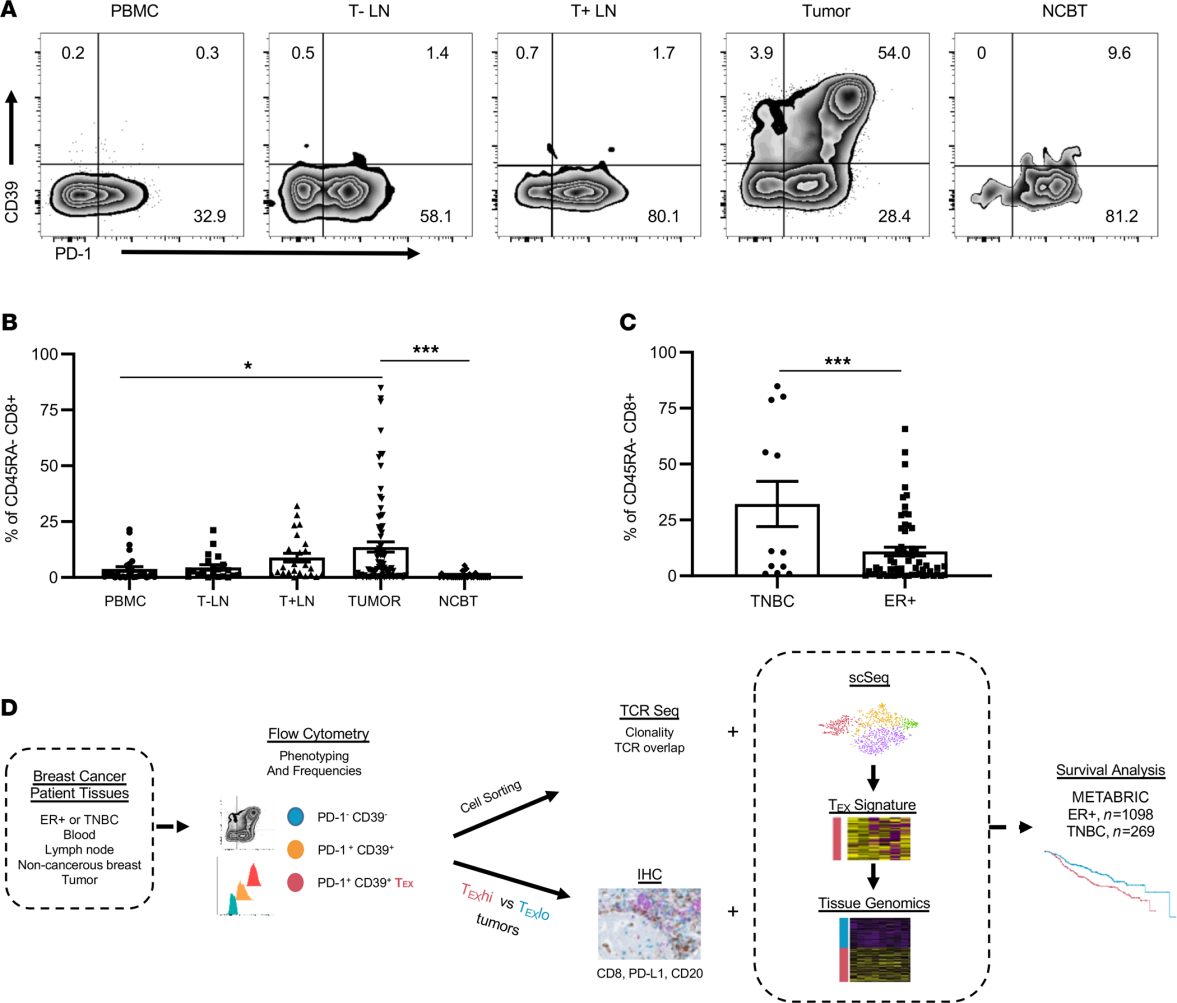
PD-1^+^CD39^+^CD8^+^ T cells in BC patient tissues. (**A**) Single-cell suspensions from peripheral blood mononuclear cells (PBMCs), tumor-negative lymph nodes (T^–^ LNs), tumor-positive lymph nodes (T^+^ LNs), tumor, and noncancerous breast tissue (NCBT) were examined by flow cytometry for expression of PD-1 and CD39 among antigen-experienced (CD45RA^–^) CD8^+^ T cells. (**B**) Frequencies of PD-1^+^CD39^+^ cells within CD45RA^–^CD8^+^ T cells in various tissues are shown (PBMC *n* = 30, T^–^ LN *n* = 21, T^+^ LN *n* = 24, tumor *n* = 77, NCBT *n* = 32). (**C**) PD-1^+^CD39^+^ frequencies are displayed within triple-negative breast cancer (TNBC) and estrogen receptor–positive (ER^+^) tumors (TNBC *n* = 11, ER^+^ *n* = 66). (**D**) Experimental workflow for analysis of CD8^+^ T cells and patient tissues. Statistics generated by 1-way ANOVA with Holm-Šídák multiple comparisons test (**B**) or unpaired Student’s *t* tests (**C** and **D**). *, *P* < 0.05; ***, *P* < 0.001.

We examined BC patient peripheral blood mononuclear cells (PBMCs), tumor-negative tumor-draining lymph nodes (T^–^ LNs), tumor-positive tumor-draining lymph nodes (T^+^ LNs), primary tumors, and noncancerous breast tissue (NCBT) by flow cytometry for the presence of CD8^+^ T cells expressing T cell exhaustion markers PD-1 and CD39 (Figure 1A; gating strategy in Supplemental Figure 1; supplemental material available online with this article; https://doi.org/10.1172/jci.insight.153963DS1). Among antigen-experienced (CD45RA^–^) CD8^+^ T cells, PD-1^+^ cells were common in all tissues, but frequencies of PD-1^+^CD39^+^CD8^+^ T cells were highest in primary tumors, followed by T^+^ LNs (Figure 1B). PD-1^+^CD39^+^CD8^+^ T cells were rarely detected in PBMCs and never in NCBT. Notably, T^+^ LNs and T^–^ LNs displayed no significant differences in frequencies of PD-1^+^CD39^+^CD8^+^ T cells. We observed high variability in the frequency of PD-1^+^CD39^+^ within CD8^+^ TILs and a higher frequency on average in TNBC tumors than ER^+^ tumors (Figure 1C). Overall, the frequency of PD-1^+^CD39^+^CD8^+^ TILs did not correlate with Ki-67 status, tumor size (pathological T status), or patient stage (Supplemental Figure 2). Higher grade ER^+^ tumors tended to have increased frequencies of PD-1^+^CD39^+^CD8^+^ TILs as compared with lower grade ER^+^ tumors, but this observation lacked statistical significance because of high interpatient variability.

**Figure 2:**
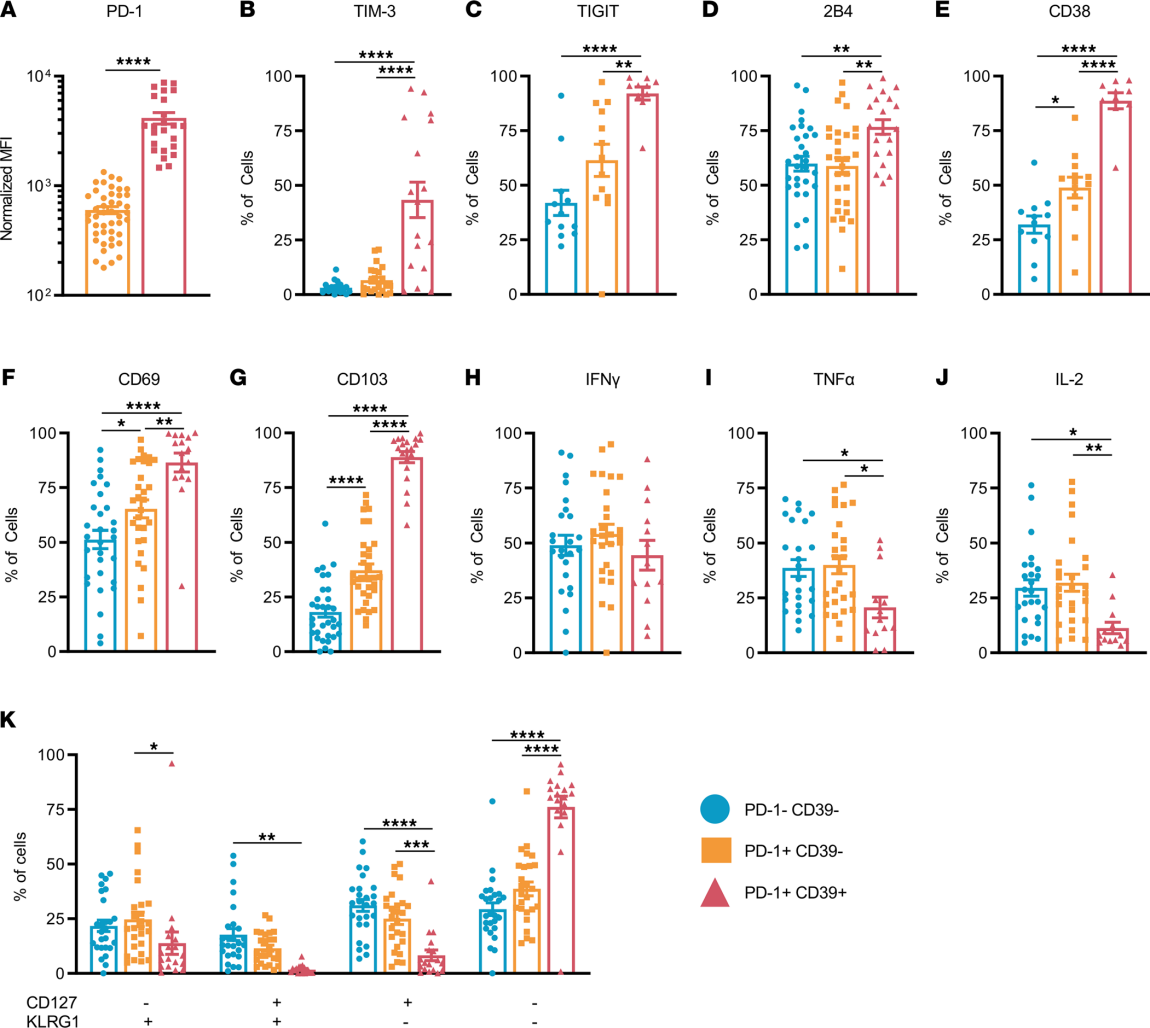
Phenotypic characterization of PD-1^+^CD39^+^CD8^+^ T cells in breast tumors. CD8^+^ TILs from patient tumors were examined by flow cytometry for expression of various proteins. (**A**) Normalized PD-1 expression of PD-1^+^CD39^–^ (orange) and PD-1^+^CD39^+^ (red) CD8^+^ TILs, calculated by subtracting MFI values of PD-1^–^CD8^+^ TILs in the same sample (*n* = 45). Frequencies of PD-1^–^CD39^–^, PD-1^+^CD39^–^, and PD-1^+^CD39^+^ (red) CD8^+^ TILs expressing (**B**) TIM-3 (*n* = 25), (**C**) TIGIT (*n* = 14), (**D**) 2B4 (*n* = 33), (**E**) CD38 (*n* = 15), (**F**) CD69 (*n* = 33), and (**G**) CD103 (*n* = 36). Frequencies of PD-1^–^CD39^–^, PD-1^+^CD39^–^, and PD-1^+^CD39^+^CD8^+^ TIL populations producing (**H**) IFN-γ (**I**) TNF-α, and (**J**) IL-2 (*n* = 29) after stimulation with PMA and ionomycin. (**K**) Frequencies of each cell population for CD127 and KLRG1 expression profiles (*n* = 30). All data were collected from 39 ER^+^ primary tumors and 6 TNBC primary tumors. Statistics generated by unpaired Student’s *t* tests (**A**) or 1-way ANOVA with Holm-Šídák multiple comparisons test (**B**–**K**). *, *P* < 0.05; **, *P* < 0.01; ***, *P* < 0.001; ****, *P* < 0.0001.

We next set out to elucidate the relationship between PD-1^+^CD39^+^CD8^+^ TILs, TME features, and patient survival using a multiomics approach (Figure 1D). Further characterization of BC patient PD-1^+^CD39^+^CD8^+^ TILs’ protein expression was performed by flow cytometry to determine if they met canonical definitions of T cell exhaustion. PD-1 levels were significantly higher on PD-1^+^CD39^+^CD8^+^ TILs relative to PD-1^+^CD39^–^CD8^+^ TILs (Figure 2A and Supplemental Figure 3A), identifying them as PD-1 “high” CD8^+^ T cells described in other tumor types (15). Relative to other CD8^+^ TILs, higher percentages of PD-1^+^CD39^+^CD8^+^ TILs expressed molecules TIM-3, T cell immunoreceptor with Ig and ITIM domains (TIGIT), 2B4, and CD38 (Figure 2, B–E, and Supplemental Figure 3, B–E). Similarly, higher percentages of PD-1^+^CD39^+^CD8^+^ TILs expressed resident memory markers CD69 and CD103 as compared with other CD8^+^ TILs (Figure 2, F and G, and Supplemental Figure 3, F and G).

We then confirmed PD-1^+^CD39^+^CD8^+^ TILs as functionally exhausted compared with other CD8^+^ TILs by examining their capacity to produce effector cytokines IFN-γ, TNF-α, and IL-2 (Figure 2, H–J, and Supplemental Figure 3H). PD-1^+^CD39^–^ and PD-1^–^CD39^–^CD8^+^ TILs displayed no differences in IFN-γ, TNF-α, or IL-2 production capacity, highlighting our previous findings that PD-1 expression alone does not identify an exhausted phenotype (18). In contrast, PD-1^+^CD39^+^CD8^+^ TILs demonstrated marked loss in production capacity of both TNF-α and IL-2, while mostly retaining IFN-γ production capacity. Such functional data formally identify PD-1^+^CD39^+^CD8^+^ TILs in human breast tumors as CD8^+^ T_EX_ with similar functional and phenotypic profiles of T_EX_ described by others in the context of other cancer malignancies and chronic disease settings (16, 17, 21, 22).

Next, we examined expression of proteins CD127 (IL-7Rα) and killer cell lectin like receptor G1 (KLRG1) to assess PD-1^+^CD39^+^CD8^+^ TILs for evidence of terminal differentiation (Figure 2K and Supplemental Figure 3I). CD127 expression is critical for homeostatic proliferation and maintenance of memory T cells, while KLRG1 expression signifies an effector T cell status (23). Loss of both CD127 and KLRG1 has been associated with a severe T cell exhaustion phenotype (24, 25). PD-1^+^CD39^+^CD8^+^ TILs primarily displayed a CD127^–^KLRG1^–^ phenotype. Comparatively, both PD-1^+^CD39^–^ and PD-1^–^CD39^–^CD8^+^ TILs contained cell populations with mixed expression of CD127 and KLRG1. Taken together this phenotyping illustrates PD-1^+^CD39^+^CD8^+^ TILs found in BC tumors as highly activated cells with both exhausted and tissue residency characteristics.

### Exhausted CD8^+^ T cells in human breast tumors are transcriptionally distinct.

**Figure 3:**
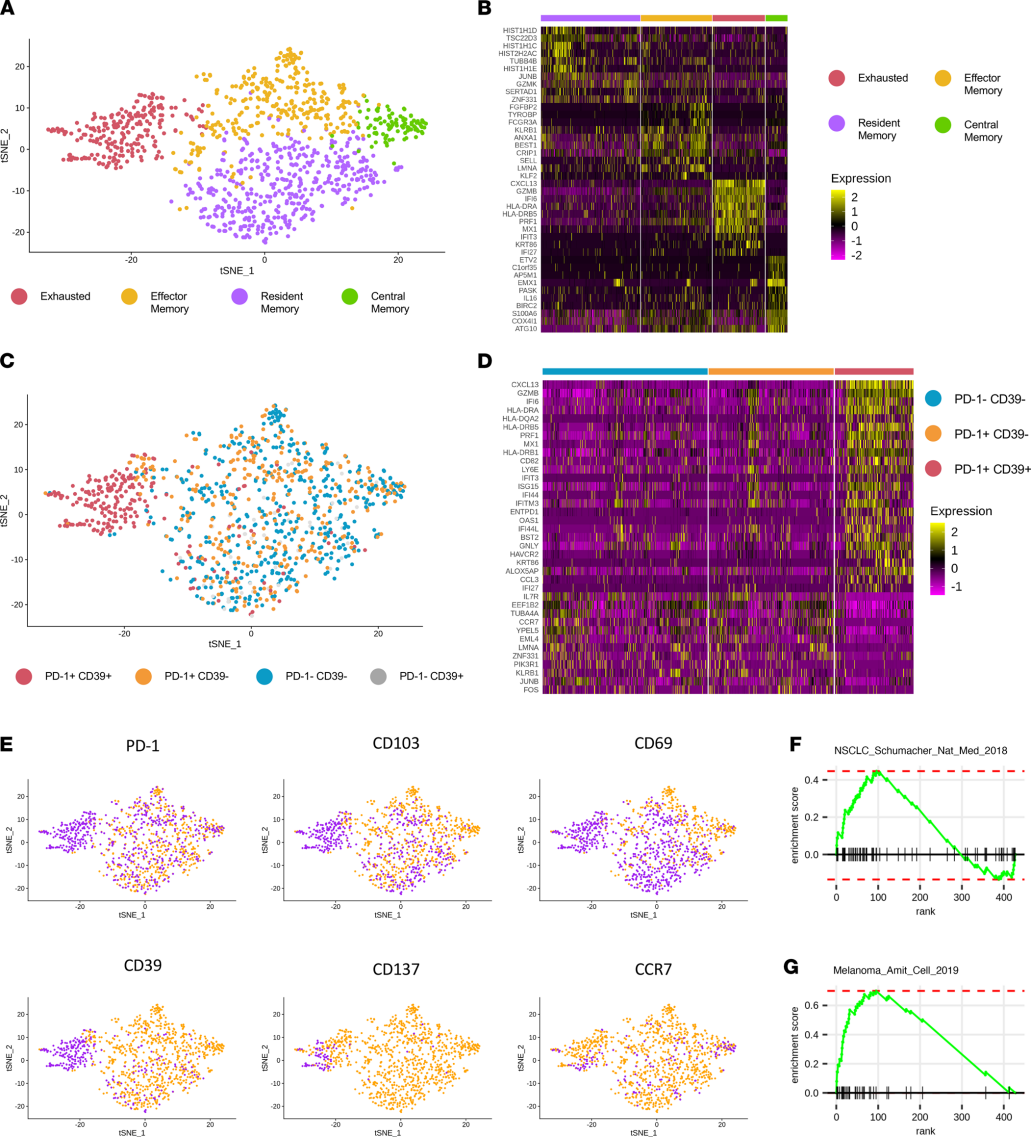
Transcriptional features of CD8^+^ T_EX_ in breast tumors. CD8^+^ T cells from 8 ER^+^ BC and 2 TNBC patient tissues were single-cell index sorted for whole-transcriptome analysis in the context of several cell surface proteins. (**A**) t-Distributed stochastic neighbor embedding (t-SNE) projection of 4 major clusters of CD8^+^ T cells identified and annotated as exhausted T cells, resident effector memory T cells, effector memory T cells, and central memory T cells. (**B**) Top 10 most differentially expressed genes for each CD8^+^ T cell cluster. (**C**) t-SNE overlay of CD8^+^ T cells identified as PD-1^–^CD39^–^ (blue), PD-1^+^CD39^–^ (orange), PD-1^+^CD39^+^ (red), or PD-1^–^CD39^+^ (gray). (**D**) Genes most significantly differentially expressed by PD-1^+^CD39^+^CD8^+^ T cells. (**E**) Overlay of cell surface protein expression onto t-SNE cluster projections. Protein expression for PD-1, CD103, CD69, CD39, CD137, and CCR7 acquired from index sort information and shown here as positively (purple) or negatively (yellow) expressed for each cell. Gene Set Enrichment Analysis (GSEA) of PD-1^+^CD39^+^CD8^+^ T cell differentially expressed genes as compared with T_EX_ signatures identified from (**F**) lung cancer and (**G**) melanoma publications. Gene rank shown is derived from the current data set. (*n* = 10 BC patients; 9 tumors; 2 T^+^LNs; 3 NCBTs; 7 matched PBMCs.)

CD8^+^ T cell exhaustion has been demonstrated as a transcriptionally and epigenetically discrete functional state in various disease settings (26–29). To assess this in the context of BC, we employed single-cell RNA sequencing of patient CD8^+^ T cells from 10 patients with BC, including 9 primary tumors, 2 T^+^ LNs, 3 NCBTs, and 7 matched PBMC samples (Supplemental Figure 4). CD8^+^ T cells stained for PD-1, CD39, CD103, CD69, CD137, and CCR7 were single-cell index sorted for downstream whole-transcriptome analysis. Unbiased Seurat cluster analysis found CD8^+^ T cells to be composed of 4 major clusters with discrete gene expression patterns (Figure 3, A and B). As expected, PD-1^+^CD39^+^ T cells occupied a unique cluster, while surprisingly PD-1^+^CD39^–^ and PD-1^–^CD39^–^ were indiscriminately found in all other T cell clusters and showed no major differences in gene expression (Figure 3, C and D).

Cell surface protein expression information collected by index sorting was then used to annotate the 4 CD8^+^ T cell clusters as T_EX_ (PD-1^+^CD39^+^), resident effector memory T cells (CD39^–^, CD103^+^, CD69^+^), effector memory T cells (CD39^–^, CD103^+/–^, CD69^+/–^), and central memory T cells (CCR7^+^) (Figure 3E). CD137 was found almost exclusively in the exhausted T cell cluster. CD103 and CD69 expression across several clusters suggests the potential acquisition of a CD103^+^CD69^+^ phenotype as T cells transition through these phenotypes. Unsurprisingly, the CCR7-expressing central memory T cell cluster was largely identified in PBMC-derived T cells.

Analysis of gene expression differences between clusters revealed several key differences between T cell populations. The central memory T cell cluster, mostly of PBMC origin, expressed genes related to proliferation capacity (*PASK*, *IL16*) and antiapoptosis (*BIRC2*), along with the memory T cell–associated gene *S100A6* (30). The activated effector memory T cell cluster expressed genes linked to T cell activation (*LMNA*, *ANXA1*), effector function (*FGFBP2*, *KLRB1*), and T cell trafficking (*SELL*, *KLF2*) (31, 32). Intriguingly, the resident effector memory T cluster demonstrated highly differentiated upregulation of histone genes and regulatory elements (*SERTAD1*, *ZNF331*) that may play a role in cell cycle regulation. The resident effector memory T cell cluster also displayed upregulated *GZMK*, in contrast with *GZMB* and *PRF1* upregulation in the exhausted T cell cluster. This observed T cell subset–specific granzyme utilization likely reflects T cell differentiation stage–specific changes in granzyme expression profiles observed by others (33). Finally, the exhausted T cell cluster showed transcriptional upregulation of genes associated with increased cytolytic activation (*GZMB*, *PRF1*), T cell activation (*HLA-DRA*), IFN response elements (*IFI6*, *MX1*, *IFI27*), and the B cell chemoattractant *CXCL13*. Additionally, we observed downregulation of activator protein 1 complex molecules *JUNB* and *FOS*; downregulation of these genes has been shown by others to mark chronically activated T_EX_ (34, 35).

We next generated an exhausted T cell gene signature composed of genes significantly elevated in T_EX_ relative to other CD8^+^ T cell populations. Log_2_ fold changes of genetic markers were considered the weights in the signature. Our exhausted T cell gene signature contains 25 genes, including *CXCL13*, *GZMB*, *IFI6*, *HLA-DRA*, *HLA-DQA2*, *HLA-DRB5*, *PRF1*, and *MX1*. The full gene signature and relative gene fold changes are shown in Supplemental Table 3. We then compared our exhausted T cell gene expression signature to exhausted T cell gene signatures produced by other groups using GSEA. We found that our exhausted T cell signature shared significant similarities to those identified from lung cancer and melanoma TILs (Figure 3, F and G) (21, 36). We also verified that our exhausted T cell gene signature had significant overlap with those produced in lymphocytic choriomeningitis virus murine models of T cell exhaustion (Supplemental Figure 5) (35, 37). Thus, the transcriptional signature of T_EX_ in patients with BC shared common features to both those seen in other disease states and classically defined T_EX_. Together our single-cell data confirm CD8^+^ T_EX_ as an activated, transcriptionally distinct CD8^+^ TIL population that has likely clonally expanded in response to cognate tumor antigens.

### Increased CD8^+^ T_EX_ are associated with IFN-γ signature–rich and immunologically distinct tumors.

**Figure 4:**
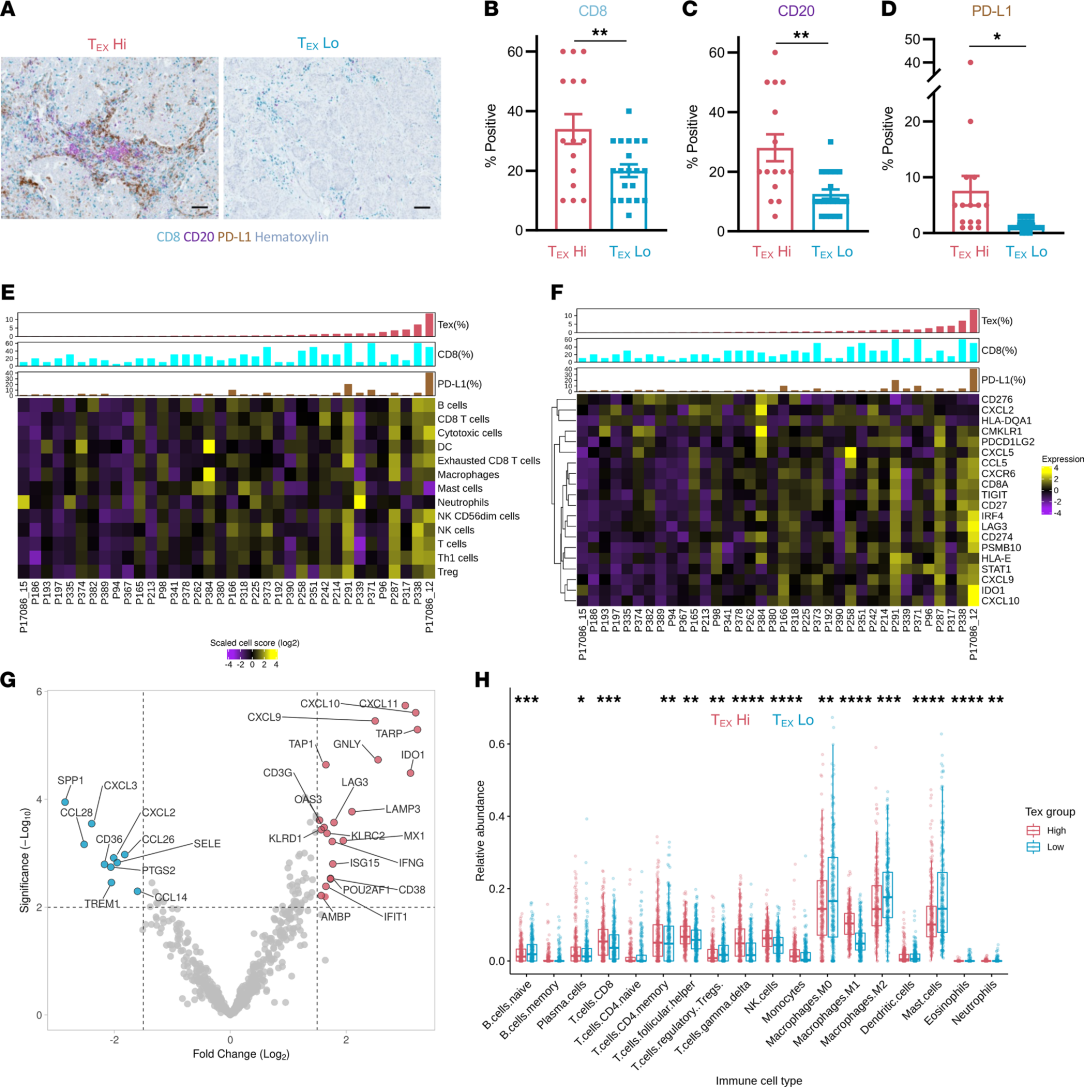
Altered immune TME in T_EX_^hi^ breast tumors. ER^+^ breast tumors defined as T_EX_^hi^ (top 50%) or T_EX_^lo^ (bottom 50%) by flow cytometry were assayed by immunohistochemistry (IHC) for CD8^+^ T cell infiltration (teal), CD20^+^ B cell infiltration (purple), and PD-L1 expression (brown). (**A**) Representative high-power fields (original magnification, 20×; scale bar: 50 μm). Clinical pathologist scoring for (**B**) CD8, (**C**) CD20, and (**D**) PD-L1 (T_EX_^hi^ *n* = 18, T_EX_^lo^ *n* = 18; unpaired Student’s *t* test). TME features of ER^+^ breast tumors were assessed by NanoString PanCancer Immune transcriptional profiling (*n* = 36). (**E**) Absolute abundance of cell type scores and (**F**) inflammation-related gene expression are displayed as heatmaps normalized across all tissues by cell type or gene (row). Tumor tissues (columns) are annotated by FACS T_EX_ frequency of CD8^+^ TILs normalized to %CD8 by IHC (%T_EX_; red), IHC CD8^+^ T cell infiltration score (%CD8; blue), and IHC PD-L1 expression score (%PD-L1; brown). (**G**) Top 30 genes differentially expressed (uncorrected Student’s *t* test *P* < 0.01) between T_EX_^hi^ and T_EX_^lo^ tumors are shown. (**H**) CIBERSORTx analysis of relative immune populations in T_EX_^hi^ (top 25%, *n* = 275) and T_EX_^lo^ (bottom 25%, *n* = 275) ER^+^ breast tumors in METABRIC database. Statistics generated by Wilcoxon’s rank-sum test. *, *P* < 0.05; **, *P* < 0.01; ***, *P* < 0.001; ****, *P* < 0.0001.

Given that CD8^+^ T_EX_ identified in BC patient tumors displayed a highly activated phenotype and largely retained the capacity to produce IFN-γ, we next asked how their presence correlated with differences in TME features. ER^+^ tumors with known fractions of CD8^+^ T_EX_ as identified by flow cytometry were curated into T_EX_ “high” (T_EX_^hi^) and T_EX_ “low” (T_EX_^lo^) tumors as defined by above and below the overall median for percentage T_EX_ of CD8^+^ TILs (8%), respectively. Pathologist assessment of CD8, CD20, and programmed death ligand 1 (PD-L1) expression was then performed on these tumors with immunohistochemistry-stained slides (Figure 4A). CD8^+^ T cell infiltration was higher in several, but not all, T_EX_^hi^ tumors (Figure 4B). In contrast, CD20^+^ B cell infiltration was higher in the majority of T_EX_^hi^ tumors (Figure 4C). Although PD-L1 expression was found to be highly variable in samples, strikingly, all tumors with stroma scoring PD-L1^+^ of 5% or higher were found in T_EX_^hi^ tumors (Figure 4D).

ER^+^ tumors with known abundance of CD8^+^ T_EX_ from flow cytometry were assessed with the NanoString nCounter PanCancer Immune Profiling Panel for immune cell composition and differential gene expression. CD8^+^ T_EX_ in tumors correlated with higher abundance of a variety of immune subsets, including B cells, overall CD8^+^ T cells, exhausted CD8^+^ T cells, Th1 cells, Tregs, and CD56^dim^ NK cells as identified by standard NanoString signatures (Figure 4E). Given the association between activated T cells, IFN-γ production, and PD-L1 expression in the TME, we assessed the expression of the previously reported “tumor inflammation signature,” which is mainly composed of IFN-γ–regulated genes (38). Several of these genes were significantly correlated with the presence of CD8^+^ T_EX_, including *CXCL10*, *IDO1*, *CXCL9*, *STAT1*, *CD274* (PD-L1), and *LAG3* (Figure 4F). Interestingly, *CD276* and *CXCL2* expression was not positively correlated with CD8^+^ T_EX_. Other genes significantly upregulated in T_EX_^hi^ tumors included *IFNG* itself, *TARP*, *GNLY*, *MX1*, and *TAP2*. Notably, genes *SPP1*, *CCL28*, *CXCL3*, *SELE*, and *CCL26* were all decreased in T_EX_^hi^ tumors (Figure 4G).

To expand on our tissue-based observations of T_EX_^hi^ and T_EX_^lo^ ER^+^ tumors, we turned to the Molecular Taxonomy of Breast Cancer International Consortium (METABRIC) public data source for gene expression analysis in a larger cohort of BC tumors (39). We designated ER^+^ tumors as T_EX_^hi^ (top 25%) and T_EX_^lo^ (bottom 25%) based on expression of our exhausted T cell gene signature derived from single-cell sequencing and performed differential gene expression analysis. T_EX_^hi^ tumors demonstrated significantly increased expression of numerous genes involved in immune surveillance and activation marked by increased expression of allograft rejection, inflammatory response, and interferon response Hallmark pathways (Supplemental Figure 6). In concurrence with NanoString analysis of our own tumor samples, these included IFN-γ signaling genes *STAT1*, *CXCL10*, and *IDO1*; antigen presentation molecules *HLA-DQA1* and *HLA-DRB1*; important T cell molecules *GZMB* and *IL7R*; and B cell–related molecules *CD79A* and *CXCL13*. Next, we utilized CIBERSORTx to interrogate differences in immune composition between T_EX_^hi^ and T_EX_^lo^ tumors by assessing relative abundance of various immune populations (40). Notably, T_EX_^hi^ tumors were composed of higher fractions of M1 macrophages, NK cells, γ/δ T cells, and CD8^+^ T cells (Figure 4H). In comparison, T_EX_^lo^ tumors were composed of higher fractions of M2 macrophages, M0 macrophages, mast cells, and naive B cells. In summary, T_EX_^hi^ tumors display an “inflamed tumor” phenotype, with upregulation of numerous IFN-γ–associated genes, increased chemokines, antigen presentation–related molecules, and antitumor immune subsets, such as M1 macrophages, NK cells, and effector CD8^+^ T cells.

### Exhausted T cell signatures denote prognostic outcome in patients with BC.

**Figure 5:**
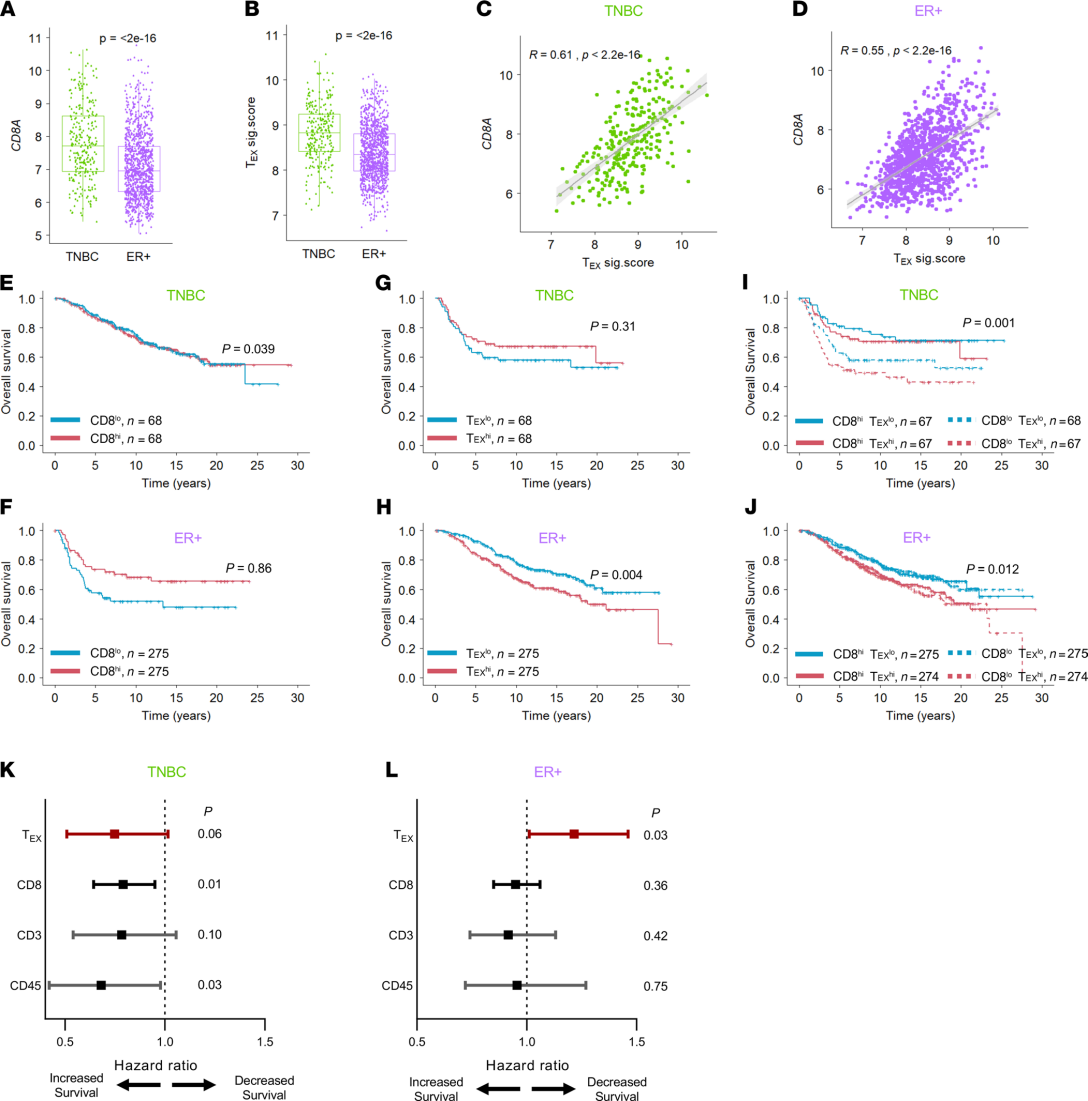
T_EX_^hi^ tumors denote decreased survival in ER^+^ BC. METABRIC tumors were used to examine (**A**) *CD8A* expression and (**B**) T_EX_ expression in TNBC (green) and ER^+^ (purple) tumors. Coexpression of *CD8A* and a T_EX_ signature in both (**C**) TNBC and (**D**) ER^+^ tumors. Tumors were stratified by *CD8A* expression into CD8^hi^ (top 25%) and CD8^lo^ (bottom 25%) groups to examine overall survival in (**E**) TNBC and (**F**) ER^+^ BC. Similarly, tumors were stratified by T_EX_ signature expression into T_EX_^hi^ (top 25%) and T_EX_^lo^ (bottom 25%) groups to examine overall survival in (**G**) TNBC and (**H**) ER^+^ BC. Tumors were further stratified into cohort quartiles as CD8^hi^T_EX_^hi^ (top 50% T_EX_ of top 50% CD8), CD8^hi^T_EX_^lo^ (bottom 50% T_EX_ of top 50% CD8), CD8^lo^T_EX_^hi^ (top 50% T_EX_ of bottom 50% CD8), and CD8^lo^T_EX_^lo^ (bottom 50% T_EX_ of bottom 50% CD8) within (**I**) TNBC and (**J**) ER^+^ tumors. Influence of T_EX_ signature on overall survival in (**K**) TNBC and (**L**) ER^+^ BC patients was compared to *CD8A*, *CD3G*, and *PTPRC* (CD45) gene expression by multivariate Cox hazard ratio assessment. Statistics generated by unpaired Student’s *t* test (**A** and **B**), nonparametric Spearman rank correlation (**C** and **D**), log-rank test (**E**–**J**), or Wald’s test (**K** and **L**).

We next aimed to unravel the relationship between CD8^+^ T_EX_, BC tumor characteristics, and patient outcomes within the METABRIC data set. As expected, increased expression of *CD8A* was found in TNBC tumors compared with ER^+^ tumors (Figure 5A). We next found signature scores for T_EX_ were higher in TNBC tumors as compared with ER^+^ tumors (Figure 5B), as also observed in our flow cytometry data. In line with these observations, PAM50 molecular classification of tumors demonstrated that exhausted T cell signatures were highest in basal tumors and slightly higher in luminal B tumors than luminal A tumors (Supplemental Figure 7A). As tumor-infiltrating T_EX_ have been shown to be specific for somatic mutation–derived neoantigens, we next investigated if increased tumor mutation burden coincided with increased T_EX_ in patients with BC (41). Surprisingly, within both TNBC and ER^+^ METABRIC cohorts, T_EX_^hi^ tumors had a statistically significant decreased number of somatic mutations detected (Supplemental Figure 7, B and C). However, the difference in mean mutation burden between T_EX_^hi^ and T_EX_^lo^ tumors was only 2 somatic variations. To confirm the lack of association between T_EX_ infiltration and high tumor mutation burden, we next performed tumor mutation load (TML) analysis on our own ER^+^ tumor tissues using a targeted TML panel. Again, observed TML did not correlate in any way with the T_EX_ frequencies of CD8^+^ TILs identified by flow cytometry (Supplemental Figure 7D). Taken together, these observations suggest that increased levels of T_EX_ CD8^+^ TILs in patients with BC cannot necessarily be accounted for by increased tumor mutation burden, although we do not discount the possibility that T_EX_ CD8^+^ TILs may be neoantigen specific.

*CD8A* expression and T_EX_ signature expression showed a modest positive correlation in both TNBC (*R* = 0.6) and ER^+^ (*R* = 0.5) METABRIC tumors, revealing that high levels of CD8^+^ T_EX_ could be found in tumors with both high and low levels of CD8^+^ T cells (Figure 5, C and D). To investigate potential divergent contributions of overall CD8^+^ T cell infiltration and CD8^+^ T_EX_ infiltration, we stratified ER^+^ and TNBC tumors into CD8^hi^ or CD8^lo^ and T_EX_^hi^ or T_EX_^lo^ based on top 25% and bottom 25% cutoffs (Supplemental Figure 7, E, F, H, and I). As expected, TNBC patients with CD8^hi^ tumors had marked increases in survival as compared with those with CD8^lo^ tumors (Figure 5E). However, in ER^+^ BC, patients with CD8^hi^ tumors and CD8^lo^ tumors demonstrated no differences in survival (Figure 5F). TNBC patients with T_EX_^hi^ (top 25%) tumors had no improved survival relative to those with T_EX_^lo^ (bottom 25%) tumors (Figure 5G). In stark contrast, ER^+^ patients with T_EX_^hi^ tumors had significantly reduced survival (Figure 5H).

We next set out to reconcile our observations regarding CD8^+^ T_EX_ and overall CD8^+^ T cell infiltration by assessing survival in the context of both variables. For survival analysis in the context of both CD8^+^ T cells and T_EX_, we further stratified tumors into 4 groups: CD8^hi^T_EX_^hi^, CD8^hi^T_EX_^lo^, CD8^lo^T_EX_^hi^, and CD8^lo^T_EX_^lo^ (Supplemental Figure 7, G and J). Patients with TNBC with CD8^hi^T_EX_^hi^ and CD8^hi^T_EX_^lo^ tumors demonstrated the best survival (Figure 5I). Strikingly, ER^+^ patients with CD8^hi^T_EX_^lo^ and CD8^lo^T_EX_^lo^ tumors demonstrated the best survival (Figure 5J). We next performed multivariate analysis of these gene signatures to confirm our observed contrast in the contribution of CD8^+^ T cell infiltration and exhausted T cell infiltration to survival in patients with TNBC and ER^+^ BC. In patients with TNBC increased overall survival was primarily driven by increased *CD8A* expression and to a lesser degree expression of T_EX_ (Figure 5K). In patients with ER^+^ BC, again survival had no association with *CD8A* expression and significantly decreased as expression of T_EX_ increased (Figure 5L). For context we compared hazard ratios to gene expression of *CD3G* and *PTPRC* (CD45). We found our exhausted T cell signature to be more predictive of outcome than immune (*PTPRC*) or T cell infiltration levels alone (*CD3G* or *CD8A*) in patients with ER^+^ BC.

Increased expression of IFN response genes has been associated with worse patient outcomes in ER^+^ BC (42). We continued to explore the relationship between tumor IFN signaling, the presence of T_EX_, and survival in patients with ER^+^ BC. In METABRIC patients with ER^+^ BC, we found a strong correlation between the IFN-γ tumor signature and our exhausted T cell signature (Supplemental Figure 8A). In comparing T_EX_^hi^ and T_EX_^lo^ ER^+^ BC tumors by differential gene expression, several of the most upregulated genes in T_EX_^hi^ tumors were involved in IFN-γ signaling and response, including *CXCL10*, *CXCL9*, *IFI27*, *IFI44*, *IFI44L*, *IFI6*, *IFIT1*, *IFIT2*, *IFIT3*, *ISG15*, *MX1*, *OAS1*, *OASL*, and *STAT1* (Supplemental Figure 8B). We then assessed these IFN-γ–associated genes within cancer cells specifically by using internal single-cell sequencing data from ER^+^ BC tumors in which we knew the fraction of T_EX_ CD8^+^ TILs as determined by flow cytometry. Again, we categorized tumors as T_EX_^hi^ and T_EX_^lo^ by being above or below the overall median for percentage T_EX_ of CD8^+^ TILs (8%). All the 14 IFN-γ–associated genes we examined, with the exception of *CXCL9*, were found to be significantly elevated in cancer cells within T_EX_^hi^ tumors (Supplemental Figure 8C). Finally, a hazard ratio analysis of patients with ER^+^ BC found a significantly increased risk for lower survival with increased tumor expression of *IFI27*, *IFI44*, *IFI44L*, *IFI6*, *IFIT1*, *IFIT2*, *IFIT3*, *ISG15*, *MX1*, *OAS1*, and *OASL* (Supplemental Figure 8D). In summary, we identify a strong connection between T_EX_ CD8^+^ TILs, IFN-γ signaling in BC cells, and reduced overall survival in patients with ER^+^ BC.

### Unfavorable survival in premenopausal ER^+^ BC patients with high exhausted T cell tumor infiltration.

**Figure 6:**
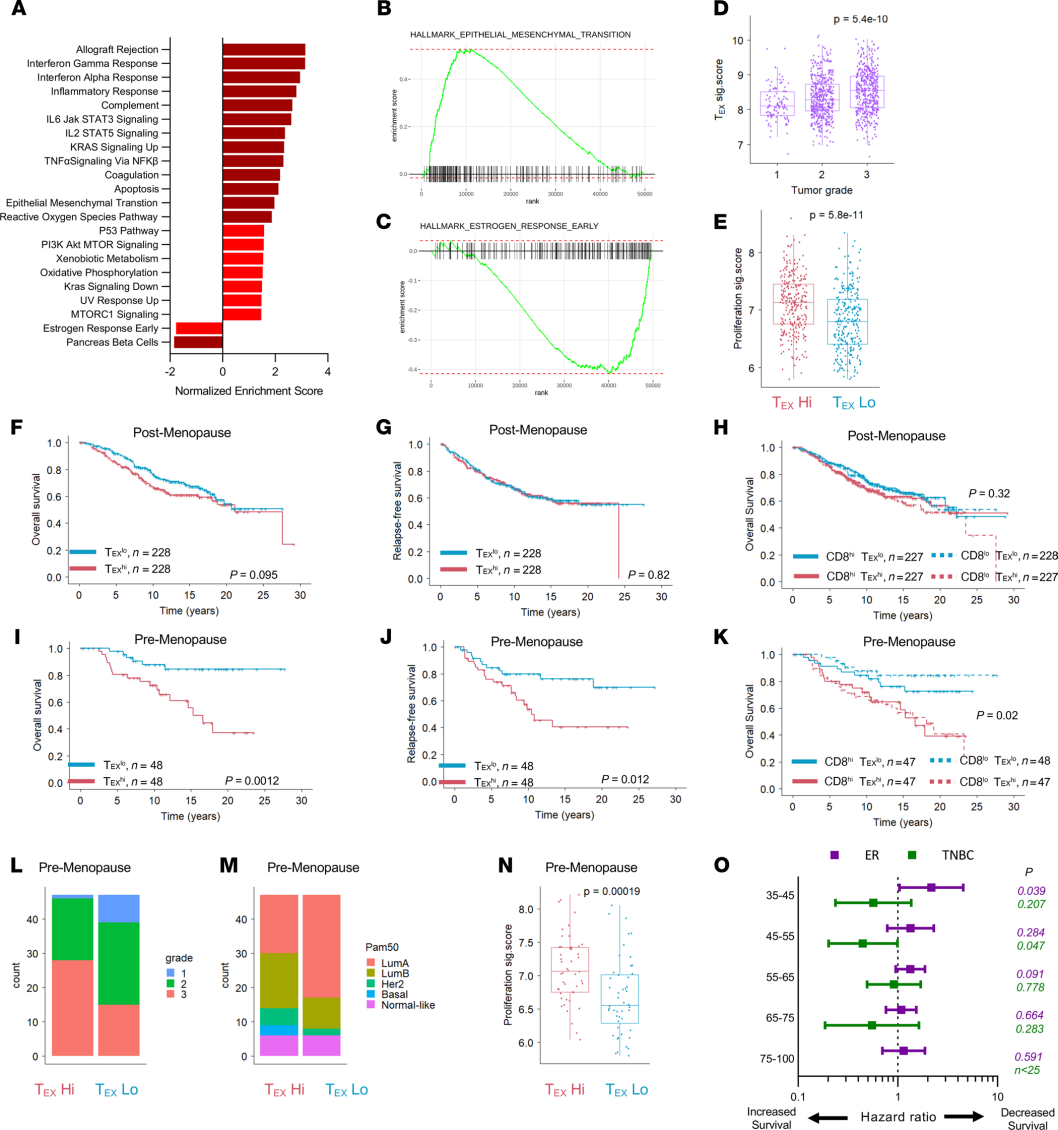
Reduced overall survival in premenopausal women with ER^+^ T_EX_^hi^ tumors. (**A**) Differential expression of Hallmark pathway gene sets between T_EX_^hi^ and T_EX_^lo^ TCGA ER^+^ BC tumors was performed. Normalized enrichment scores are shown for all pathways with *P* < 0.05. Gene set enrichment for genes upregulated in T_EX_^hi^ tumors are shown for (**B**) epithelial-mesenchymal transition and (**C**) estrogen response early pathways. (**D**) METABRIC ER^+^ tumors were assessed for the T_EX_ signature among tumor grades. (**E**) T_EX_^hi^ and T_EX_^lo^ tumors were compared for a proliferation signature score. METABRIC-defined postmenopausal ER^+^ patient tumors were stratified by T_EX_ signature expression into T_EX_^hi^ (top 25%) and T_EX_^lo^ (bottom 25%) groups to examine (**F**) overall survival and (**G**) relapse-free survival. (**H**) Overall survival was also compared between CD8^hi^T_EX_^hi^ (top 50% T_EX_ of top 50% CD8), CD8^hi^T_EX_^lo^ (bottom 50% T_EX_ of top 50% CD8), CD8^lo^T_EX_^hi^ (top 50% T_EX_ of bottom 50% CD8), and CD8^lo^T_EX_^lo^ (bottom 50% T_EX_ of bottom 50% CD8). In the same way, (**I**) overall survival and (**J**) relapse-free survival were compared in premenopausal ER^+^ patient T_EX_^hi^ and T_EX_^lo^ groups. (**K**) Overall survival in premenopausal CD8^hi^T_EX_^hi^, CD8^hi^T_EX_^lo^, CD8^lo^T_EX_^hi^, and CD8^lo^T_EX_^lo^ subgroups. Premenopausal ER^+^ BC patient tumor (**L**) grade, (**M**) PAM50 molecular subset, (**N**) and proliferation signature score in T_EX_^hi^ and T_EX_^lo^ tumors. (**O**) Multivariate Cox hazard ratios for overall survival in relation to T_EX_ signature expression among varying age groups in ER^+^ and TNBC METABRIC patients. Statistics generated as noted or by 1-way ANOVA with Holm-Šídák multiple comparisons *t* test (**D**), by unpaired Student’s *t* test (**E** and **N**), or by log-rank test (**F**–**K**).

To further dissect features of T_EX_^hi^ ER^+^ BC patient tumors, we examined The Cancer Genome Atlas (TCGA) repository data to validate and expand on our findings in the METABRIC cohort. Hallmark pathway analysis similarly found increased expression of several immune-related pathways in ER^+^ tumors, including allograft rejection, interferon responses, and inflammatory responses (Figure 6A). Intriguingly, our analysis of T_EX_^hi^ ER^+^ tumors also identified increased expression of genes related to epithelial-mesenchymal transition and decreased expression of early estrogen response genes, potentially suggesting an association between T_EX_ CD8^+^ TILs and more aggressive tumor features (Figure 6, B and C). Indeed, within the ER^+^ METABRIC cohort, we found that exhausted T cell signatures generally increased in ER^+^ tumors as the grade of the tumor increased, although high expression of T_EX_ was still identified in both grade 1 and grade 2 tumors (Figure 6D). Furthermore, exhausted T cell signatures were increased in ER^+^ tumors with either Basal or Luminal B PAM50 subclassification and tended to have diminished progesterone receptor expression (Supplemental Figure 9, A and B). Importantly, we also found that as compared with T_EX_^lo^ ER^+^ tumors, T_EX_^hi^ ER^+^ tumors had a significantly increased proliferation signature (Figure 6E). No associations were identified between the presence of T_EX_ CD8^+^ TILs, patient stage, tumor size, menopause state, or age (Supplemental Figure 9, C–F).

Given the findings of a more aggressive tumor phenotype in T_EX_^hi^ ER^+^ tumors, including decreased estrogen response–related gene expression, we hypothesized that survival characteristics may be different in premenopausal and postmenopausal patients with ER^+^ BC. Using the menopausal status as defined by METABRIC (cutoff of 50 years old), we separately examined overall survival and relapse-free survival in premenopausal and postmenopausal patients with ER^+^ BC. In postmenopausal women, overall survival trended to be reduced in patients with T_EX_^hi^ tumors (Figure 6F). However, no significant differences in relapse-free survival were found (Figure 6G). Similarly, dividing postmenopausal tumors into CD8^hi^T_EX_^hi^, CD8^hi^T_EX_^lo^, CD8^lo^T_EX_^hi^, and CD8^lo^T_EX_^lo^ did not distinguish significant survival differences (Figure 6H). On the other hand, premenopausal women with T_EX_^hi^ tumors had dramatically reduced overall survival and relapse-free survival as compared with premenopausal women with T_EX_^lo^ tumors (Figure 6, I and J). Further analysis of CD8^hi^T_EX_^hi^, CD8^hi^T_EX_^lo^, CD8^lo^T_EX_^hi^, and CD8^lo^T_EX_^lo^ groups showed again that diminished survival was strictly associated with a T_EX_^hi^ phenotype, regardless of being CD8^hi^ or CD8^lo^ (Figure 6K). As clinical presentation of premenopausal and postmenopausal women may vary, we repeated and validated our survival findings in grade 1 and 2 only ER^+^ patients and stage 4–excluded ER^+^ patients (Supplemental Figure 10). Within premenopausal patients, T_EX_^hi^ tumors were increasingly composed of higher grade and Luminal B, HER2^+^, and basal molecular subset tumors, highlighting heterogeneity in the features of T_EX_^hi^ tumors (Figure 6, L and M). More striking was a highly increased proliferation signature in premenopausal T_EX_^hi^ tumors as compared with premenopausal T_EX_^lo^ tumors (Figure 6N). A hazard ratio analysis of survival risk imparted by our T_EX_ signature identified women aged 35–45 as a group with the lowest overall survival and that survival risk associated with T_EX_ steadily declined with age (Figure 6O). Together these findings connect significantly reduced survival in younger premenopausal women that can be defined by high infiltration of T_EX_ CD8^+^ TILs.

### High expression of an exhausted T cell signature identifies high-risk premenopausal patients with intermediate Oncotype DX Breast Recurrence Scores.

Additional biomarkers to guide clinical care of patients with early-stage ER^+^ BC are needed. Gene expression testing to evaluate risk of recurrence is now standard of care for patients with early-stage BC. A prominent example of such gene expression testing is the Oncotype DX Breast Recurrence Score (BRS). Currently, treatment strategies for ER^+^ BC patients with intermediate BRSs are less clear. With this in mind, we next hypothesized that added stratification of ER^+^ tumors by our T_EX_ signature could provide additional prognostication value in the context of patients with intermediate Oncotype DX BRS.

Without exact BRSs available, we utilized the gene expression of the Oncotype DX 21-gene assay to calculate a BRS signature (43). T_EX_ signature expression and relative BRS were weakly associated (*R* = 0.3) in all, postmenopausal only, or premenopausal only METABRIC ER^+^ patients (Supplemental Figure 11). We did, however, observe that T_EX_^hi^ tumors appeared to be a subset of tumors with an intermediate BRS, suggesting that the T_EX_ signature could be used to further segregate these patients into distinct survival outcomes.

**Figure 7:**
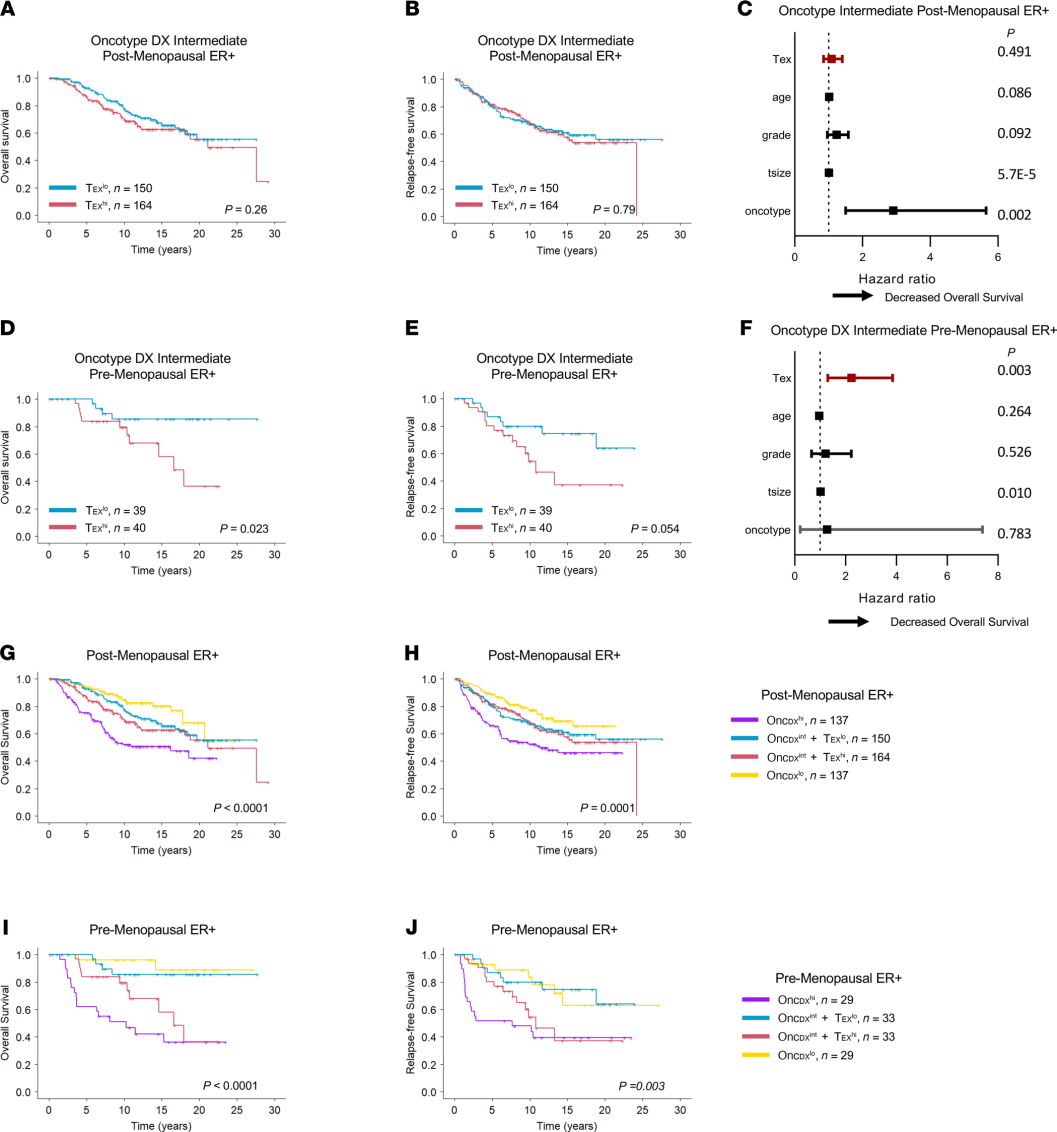
A T_EX_^hi^ signature identifies reduced survival in premenopausal patients with intermediate Oncotype DX BRSs. A relative Oncotype DX BRS was calculated for METABRIC ER^+^ tumors. Tumors were classified as Oncotype DX BRS high (top 15%), low (bottom 15%), and intermediate (middle 70%). Postmenopausal patients within intermediate Oncotype DX BRS (Onc_DX_^int^) patients were further stratified as T_EX_^hi^ (top 25%) and T_EX_^lo^ (bottom 25%) and examined for differences in (**A**) overall survival, (**B**) relapse-free survival, and (**C**) influence on overall survival by multivariate Cox hazard ratio assessment. Similarly, premenopausal patients within Onc_DX_^int^ patients were further stratified as T_EX_^hi^ and T_EX_^lo^ and examined for differences in (**D**) overall survival, (**E**) relapse-free survival, and (**F**) influence on overall survival by multivariate Cox hazard ratio assessment. Overall survival and relapse-free survival were then compared among postmenopausal (**G** and **H**) and premenopausal (**I** and **J**) ER^+^ patients between those defined as Onc_DX_^hi^, Onc_DX_^int^ + T_EX_^lo^, Onc_DX_^int^ + T_EX_^hi^, and Onc_DX_^lo^. Statistics generated by log-rank test (**A**, **B**, **D**, **E**, and **G**–**J**). Cox hazard ratios were calculated using multivariate accounting for variables shown (**C** and **F**).

To investigate this, we next defined METABRIC ER^+^ patients as having a high (top 15%) Oncotype DX BRS (Onc_DX_^hi^), intermediate (middle 70%) Oncotype DX BRS (Onc_DX_^int^), or low (bottom 15%) Oncotype DX BRS (Onc_DX_^lo^) based on observable distributions among patients and published frequencies of these clinical phenotypes (44). Within postmenopausal Onc_DX_^int^ patients, T_EX_^hi^ and T_EX_^lo^ tumors did not display significant differences in overall survival or relapse-free survival (Figure 7, A and B). Additionally, multivariate analysis did not show any significant influence of T_EX_ signature expression on overall survival (Figure 7C). In contrast, within premenopausal Onc_DX_^int^ patients, patients with T_EX_^hi^ tumors demonstrated significantly reduced overall survival and relapse-free survival as compared with those with T_EX_^lo^ tumors (Figure 7, D and E). Multivariate analysis further showed that increased T_EX_ signature expression significantly associated with decreased overall survival and more so than patient age, tumor grade, tumor size, or even Oncotype DX BRS in premenopausal Onc_DX_^int^ patients (Figure 7F).

We next performed a stepwise model selection to prune the multivariate analysis to estimate hazard ratios for survival (Supplemental Figure 12). To ensure that the influence of our T_EX_ signature was associated with survival independently of overall immune and T cell infiltration, we also included gene expression of *CD8A*, *CD3G*, and *PTPRC* in addition to patient age, tumor grade, tumor size, and LN status. Of all variables, T_EX_ was found to be the most influential for both overall survival and relapse-free survival.

For overall survival, each additional unit of T_EX_ signature was associated with a 91% (*P* value = 0.03) increase in risk for Onc_DX_^int^ patients. For relapse-free survival, each additional unit of T_EX_ signature was associated with a 76% (*P* value = 0.01) for Onc_DX_^int^ patients.

Our findings demonstrated the value of using our T_EX_ signature to further prognosticate Onc_DX_^int^ patients. We therefore next examined patient survival characteristics in the context of 4 subgroups distilled from Oncotype DX BRS and T_EX_ signature expression: Onc_DX_^hi^, Onc_DX_^int^ + T_EX_^lo^, Onc_DX_^int^ + T_EX_^hi^, and Onc_DX_^lo^. In postmenopausal patients, Onc_DX_^hi^ patients and Onc_DX_^lo^ patients had the shortest and longest survival outcomes, respectively, but Onc_DX_^int^ + T_EX_^hi^ patients did not demonstrate significantly different survival characteristics from Onc_DX_^int^ + T_EX_^lo^ patients (Figure 7, G and H). In premenopausal patients, Onc_DX_^hi^ patients again had the shortest survival outcomes, but Onc_DX_^int^ + T_EX_^hi^ patients demonstrated dramatically decreased survival characteristics as compared with Onc_DX_^int^ + T_EX_^lo^ patients (Figure 7, I and J). Surprisingly, Onc_DX_^int^^+^ T_EX_^lo^ patients had remarkably similar survival characteristics to Onc_DX_^lo^ patients. These findings establish a T_EX_ signature as a useful means to further segregate high-risk and low-risk patients within premenopausal Onc_DX_^int^ patients.

## Discussion

In this study, we showed that CD8^+^ T_EX_ occur within a subset of human breast tumors. These CD8^+^ T_EX_ were identified by distinct phenotypic properties, including PD-1 and CD39 coexpression, increased checkpoint molecule expression, reduced CD127 expression, and reduced cytokine production capacity. Single-cell sequencing revealed that CD8^+^ T_EX_ in patients with BC are transcriptionally unique. Furthermore, we showed that increased presence of CD8^+^ T_EX_ occurs in immunologically distinct tumors with increased expression of IFN-γ–related genes, including PD-L1. We showed that despite signs of increased immune activation, ER^+^ BC patients with a high CD8^+^ T_EX_ signature experienced decreased survival. Survival was found to be most dramatically reduced in premenopausal patients with ER^+^ BC, identifying an important connection between antitumor immunity and menopausal status. Last, we demonstrated the clinical utility in using our T_EX_ signature to identify premenopausal Onc_DX_^int^ patients with decreased survival outcomes.

BC tumors, which predominantly have a low mutation burden, are widely viewed as nonimmunogenic (45). We found that CD8^+^ T_EX_ can be identified in a subset of both TNBC and ER^+^ breast tumors, suggesting tumor antigen recognition even in ER^+^ tumors. Phenotyping of these T_EX_ corroborates robust activation and clonal expansion in response to antigen. Tumor immunogenicity is generally thought to be correlated with increased tumor mutation burden and the resulting neoantigen-driven T cell reactivity to cancer cells (46, 47). Indeed, tumor neoantigen–specific and exhausted CD8^+^ T cells have been described (48). Somewhat surprisingly, we did not find a correlation between the presence of CD8^+^ T_EX_ TILs and tumor mutation burden. These findings do not preclude the possibility that CD8^+^ T_EX_ in patients with BC are neoantigen specific and highlight the need for further work to dissect antigen specificity in BC.

Our data connect the presence of CD8^+^ T_EX_ with an IFN-γ–rich TME and reduced survival in patients with ER^+^ BC. Other groups have also found evidence for immune activation in ER^+^ breast tumors. Wagner et al. showed that increased frequencies of PD-1^+^CD38^+^CD8^+^ T cells, which we demonstrate as CD8^+^ T_EX_, correlate with the presence of PD-L1^+^ tumor-associated macrophages in high-grade ER^+^ tumors (49). Mirroring our CD8^+^ T_EX_ signature analysis, Thorsson et al. demonstrated IFN-γ signature–enriched tumors to be most frequent in TNBC, followed by luminal B BC and then luminal A BC (50). We find that increased T_EX_ associated with reduced overall survival in patients with ER^+^ BC but not TNBC. This further depicts ER^+^ BC and TNBC as having substantially different features of immune-cancer interaction. Standard-of-care therapy, average time to relapse, and cancer cell biology are clearly different between these BC subsets and may play a role in these differential outcomes (51). Further studies exploring the development of CD8^+^ T_EX_ in the context of BC neoadjuvant chemotherapy and adjuvant therapy regimens may reveal mechanisms for the survival characteristic differences between patients with ER^+^ BC and TNBC.

Increased IFN-γ in the TME is generally considered to reflect an active antitumor immune response beneficial to patient outcomes. However, we demonstrate that high levels of CD8^+^ T_EX_ and IFN-γ denoted poor outcomes in patients with ER^+^ BC, most significantly in premenopausal patients in which circulating estrogen levels are highest (52). Our evidence therefore lends support to other findings of the potential protumorigenic role of IFN-γ signaling (53). In support of this, increased expression of IFN response genes and JAK/STAT signaling genes has been found in both chemotherapy- and tamoxifen-resistant ER^+^ tumors (42, 54, 56). Additionally, phosphorylated STAT1 has been shown to be increased in premenopausal ER^+^ BC patients with worse survival (56). Although the exact mechanism for the relationship between increased tumorigenesis and IFN signaling in ER^+^ tumors is not yet clear, there is evidence that STAT1 and ER signaling may synergize for enhanced cancer cell proliferation (57). Our results suggest that tumor-infiltrating CD8^+^ T_EX_ may therefore inadvertently yield a rich source of IFN-γ in the TME that is protumorigenic and prometastatic in the context of ER^+^ BC tumors. We demonstrate evidence for this by finding strong correlations between T_EX_, increased tumor proliferation, increased tumor grade, and decreased survival in patients with ER^+^ BC. A novel therapeutic strategy that targets IFN signaling may be a viable approach for T_EX_^hi^ ER^+^ BC (58).

Standard of care for patients with early-stage ER^+^ BC is currently guided by hormone receptor expression, pathological tumor features, and genomic testing, such as the Oncotype DX BRS (59). Patients with high Oncotype DX BRSs have significantly increased risk of relapse and benefit from chemotherapy intervention (43). However, treatment strategies for approximately 70% of ER^+^ BC patients with intermediate Oncotype DX BRSs are less clear, and clinical outcomes differ further between premenopausal and postmenopausal women (60). Here, we demonstrate the use of T_EX_ gene signature to further select for high-risk patients among those categorized as premenopausal and Onc_DX_^int^. Our results suggest that early-stage premenopausal Onc_DX_^int^ T_EX_^hi^ patients are a high-risk cohort that may benefit from adjuvant or neoadjuvant therapies. Future preclinical and clinical studies are needed to dissect the relationship between CD8^+^ T_EX_, metastasis development, and response to therapy in the context of both hormone receptor status and menopausal status.

## Methods

### Human samples.

Tissues were obtained from consented patients with BC undergoing standard-of-care therapy at City of Hope. Patient characteristics are summarized in Supplemental Table 1. Classification of tumor samples as ER^+^, progesterone receptor–positive (PR^+^), or HER2^+^ was performed by clinical pathologists. NCBTs were composed of tissue from high-risk patient prophylactic mastectomies, contralateral breast from BC patient mastectomies, or tumor-adjacent tissue. Due to limited cell numbers obtained from patient tumor samples, not all analyses shown were performed on the same samples. Tissue samples were provided by the City of Hope Biospecimen Repository, which is funded in part by the National Cancer Institute (NCI). Other investigators may have received specimens from the same patients.

### Sample processing.

Patient peripheral blood was obtained by venipuncture using heparin collection tubes, transported at room temperature from the clinic to the lab, and processed within 6 hours of drawing. PBMCs were isolated via Ficoll-Paque separation (GE Healthcare, now Cytiva) following the manufacturer’s instructions. Solid tissue specimens were collected by surgical resection and collected in tubes containing cold HBSS (Life Technologies, Thermo Fisher Scientific) and transported on ice to the laboratory for processing within 1 hour of surgery. T^–^ LNs were mechanically dissociated and filtered into single-cell suspensions. Tumor, T^+^ LNs, and NCBTs were minced into pieces; mechanically dissociated with a gentleMACS Dissociator (Miltenyi Biotec); and enzymatically treated with 0.2 Wunsch U/mL Liberase TM (Roche) and 10 U/mL DNase (MilliporeSigma) in RPMI for up to 1 hour as needed. If necessary, RBC lysis was performed using RBC Lysis Buffer (BioLegend).

### Flow cytometry.

Single-cell suspensions were stained at room temperature in 2% FBS in PBS. For cytokine production assays, cells were stimulated with 50 ng/mL PMA (MilliporeSigma) and 1 μg/mL ionomycin (MilliporeSigma) in the presence of GolgiPlug (BioLegend) for 4 hours. Overnight fixation was performed as needed with IC Fixation Buffer (eBioscience, Thermo Fisher Scientific). Fixation and permeabilization were performed with BD Biosciences Cytofix/Cytoperm buffers for intracellular cytokine staining. Antibody cocktails were diluted in Brilliant Violet Buffer (BD Biosciences) when using 2 or more Brilliant Violet–labeled antibodies. Samples were acquired using a BD Biosciences Fortessa operating FACSDiva 6.1.3. Photomultiplier tube voltages were set using BD Biosciences CS&T Beads. Compensation was calculated using single-stained OneComp compensation beads (eBioscience, Thermo Fisher Scientific). Samples were stained with fluorescently tagged antibodies detailed in Supplemental Table 2. Antibodies were titrated for optimal signal-to-noise ratio prior to use. Flow cytometry analysis was performed using FlowJo v10.6. All samples were gated on single cells, lymphocytes, and CD3^+^CD8^+^ populations (Supplemental Figure 1). Histograms and zebra plots are used to display data.

### T cell single-cell sequencing and analysis.

CD8^+^ T cells were stained with monoclonal antibodies against CD8, PD-1, CD39, CD103, CD69, CD137, and CCR7 as described above and then single-cell index sorted using a FACSAria III system into Precise WTA 96-well plates (both from BD Biosciences) for whole-transcriptome analysis. Single-cell libraries were prepared as recommended by the manufacturer. Sequencing was performed on an Illumina HiSeq 2500 with an estimated 250,000 reads per cell. The raw counts were imputed by scImpute R package (v0.0.7) with kcluster = 5 (61). The imputed counts were analyzed by Seurat R package (v3.1.4) (62). Briefly, nonviable cells (defined as the cells in which more than 20% expressing genes are mitochondrial genes) were removed (Supplemental Methods A). In addition, the potential empty well or duplets (defined as cells in which fewer than 200 genes are expressed or more than 2500 genes are expressed, respectively) were also discarded for further analysis (Supplemental Methods B). The normalization was implemented by Seurat with default settings. The top 1000 most variable protein-encoding genes were selected for principal component analysis (PCA; Supplemental Methods C). Based on the 2 heuristic methods in Seurat (modified Jack Straw procedure and ranking variance method, Supplemental Methods, D and E), the top 12 principal components were used for further nonlinear dimensional reduction (i.e., t-SNE) and clustering analysis. Four major T cell clusters were found and visualized by t-SNE projection. Based on the FACS markers and genetic markers, all the T cell clusters were annotated. The signature of T_EX_ was filtered from the genetic markers based on the log_2_ fold changes (>1.0, i.e., larger than 2-fold change compared with the other T cells) and adjusted *P* value (<0.10). Single-cell sequencing data of T cells are deposited under the National Center for Biotechnology Information’s Gene Expression Omnibus (GEO) accession number GSE190202.

### Tumor single-cell sequencing and analysis.

Tumor single-cell RNA sequencing was implemented through 10x Genomics Chromium platform with recommended procedures. CellRanger was used to align sequence reads to the human genome and count the aligned transcripts for each cell. The raw counts for each tumor sample were directly filtered, normalized, and scaled by Seurat R package (v3.1.4) with the same parameters described above. The top 2000 most variable genes were selected for sample integration and PCA. All the tumor samples were integrated with the standard integration workflow in Seurat (i.e., “FindIntegrationAnchors” and “IntegrateData” functions in Seurat with 50 dimensionality). PCA was implemented for the integrated data object. Based on the 2 heuristic methods in Seurat (modified Jack Straw procedure and ranking variance method), the top 20 principal components were used for further nonlinearly dimensional reduction (i.e., uniform manifold approximation and projection) and clustering analysis. The clusters with PTPRC^–^EPCAM^+^ cells were annotated as tumor cell clusters. The tumor cells were stratified into T_EX_^hi^ and T_EX_^lo^ groups based on the T_EX_ abundance with FACS. The normalized gene expression of genes of interest in tumor cells was compared between T_EX_^hi^ and T_EX_^lo^ groups with Wilcoxon’s rank-sum test. Single-cell sequencing data of T cells are deposited under the GEO accession number GSE190202.

### Public genomic data analysis.

To evaluate the prognosis effect of T_EX_ in BC, METABRIC, one of the largest BC multiomics databases, was downloaded with the latest clinical information and normalized expression data (Illumina HT 12 platform) from European Genome-Phenome Archive (data set IDs EGAD00010000210 and EGAD00010000211) (39, 63). A total of 1992 patients’ data were obtained and organized. Based on the hormone receptor statuses, the ER^+^ (all ER^+^ patients) and TNBC (ER^–^PR^–^HER2^–^) populations were stratified as 1098 and 269 patients, respectively. The signature scores of T_EX_ and other signatures were calculated using the sig.score function in genefu R package (v2.18.1) with default settings (64). The log_2_ fold changes of genetic markers were considered as the weights in the signature. Based on the T_EX_ score and *CD8A* expression, the ER^+^ and TNBC cohorts were stratified as described in figure legends and text. The survival comparison and Kaplan-Meier curves between groups were implemented by survminer (v0.4.8) and survival (v3.2-7) R packages with log-rank statistics. Hazard ratios were generated with a Cox proportional-hazards model in univariate or multivariate plots. The multivariate Cox regression analysis accounted for influence of age, tumor grade, tumor size, and nodal status or as described. The association between T_EX_ signature scores and molecular and pathological features was investigated by R (v3.6.2). A tumor proliferation score was calculated based on the expression of a 19-gene proliferation signature (65).

The gene expression data (including counts and FPKM-UQ) of BC primary tumor samples of TCGA was downloaded from NCI Genomic Data Commons data portal with the corresponding clinical information (including hormone receptor statuses and overall survival). Based on the hormone receptor statuses, 538 ER^+^ patients with primary tumors were found. The same approach described above was used to calculate the T_EX_ signature scores for TCGA-BRCA ER^+^ primary tumors with normalized expression data (FPKM-UQ). The ER^+^ patients were further stratified into T_EX_^hi^ and T_EX_^lo^ cohorts with the top 25% highest T_EX_ signature scores and bottom 25% lowest T_EX_ signature scores, respectively. The differential expression analysis for T_EX_^hi^ and T_EX_^lo^ groups was implemented by DESeq2 R package (v1.30.0) with the count data and recommended normalization procedure. The significantly differentially expressed genes were defined as the genes with adjusted *P* < 0.05 and log_2_ fold change < –1 or > 1. These genes were further used to implement GSEA with the Hallmark pathway sets (msigdbr package, v7.2) with fgsea R package (v1.16.0) (66). The fgsea function was used with log_2_ fold changes as the rank score (stats), 1000 permutations, and other recommended settings.

CIBERSORTx was used to deconvolute the METABRIC expression data to 22 major immune cell types (LM22 signature) with standard data preprocessing procedure and 500 permutations for significance analysis (40, 67). The relative abundances of all the 22 major immune cell types between T_EX_^hi^ and T_EX_^lo^ cohorts were compared by Wilcoxon’s signed-rank test. The differential expression analysis between T_EX_^hi^ and T_EX_^lo^ cohorts of METABRIC expression data was implemented by limma R packages (v3.42.2) and visualized by EnhancedVolcano R package (v1.4.0) (68). The GSEA was implemented by GSEA software (v4.0.3) with the latest Hallmark Molecular Signatures Database from Broad Institute (66, 69).

The in-house Oncotype DX scores for all the METABRIC ER^+^ breast tumors (*n* = 1098) were calculated using the “sig.score” function in genefu R package (v2.18.1) with default settings. The weights of genes in Oncotype DX signature were calculated based on the Recurrence Score algorithm described in Paik et al. (43). The standard Oncotype DX scores (0–100, Oncotype DX BRS) were calculated using the “oncotypedx” function in genefu R package (v2.18.1) with default settings (60). A strong correlation between in-house and standard Oncotype DX scores was observed (*r* = 0.89, *P* < 0.01), and the stratification criteria for standard Onc_DX_^hi^ and Onc_DX_^lo^ groups were aligned with 85% (scaled score > 25) and 15% (scaled score < 15) percentiles of the whole population of ER^+^ BC patients (44). As in-house Oncotype DX scores were strongly correlated with the standard Oncotype DX scores and had a more similar scale to T_EX_ scores, the in-house Oncotype DX scores were used for the following analysis. Based on the T_EX_ score and in-house Oncotype DX scores, the ER^+^ cohort was stratified as described in figure legends and text. The survival comparison and analysis were implemented with the same statistics and packages for CD8A expression (details described above).

### NanoString gene expression analysis.

RNA was extracted from 10 μm thick slices of unbaked FFPE tissue using QIAGEN miRNeasy FFPE kits. RNA transcripts were detected using PanCancer Immune Panel with nCounter technology (NanoString Technologies). RNA concentration was assessed with the NanoDrop spectrophotometer ND-1000 and Qubit 3.0 Fluorometer (Thermo Fisher Scientific). RNA fragmentation and quality control were further determined by 2100 Bioanalyzer (Agilent). Total RNA was hybridized overnight at 65°C for 14 to 18 hours as per manufacturers’ recommendations. After hybridization, the probe-target mixture was purified by nCounter Prep Station and then quantified with nCounter Digital Analyzer (NanoString Technologies). Quality control and normalization of data were performed with nSolver Analysis Software version 4.0 (NanoString Technologies), and the measured gene expression values were normalized to the geometric mean of 40 housekeeping genes. Advanced analysis was conducted using nCounter Advanced Analysis Software version 2.0.115. Heatmaps were generated using the ComplexHeatmap R package (v2.1.1). Volcano plots were generated using the VolcaNoseR Shiny app (https://goedhart.shinyapps.io/VolcaNoseR/). NanoString genomic data are deposited under the GEO accession number GSE190169.

### TML assessment.

DNA was extracted from 10 μm thick slices of unbaked FFPE tissue using QIAGEN QIAamp DNA FFPE Tissue kits and measured using Qubit 3.0 Fluorometer (Thermo Fischer Scientific). Using 20 ng of DNA, the library was prepared following manufacturers’ instructions. Once the libraries were generated, concentration was measured by quantitative PCR (qPCR) using the Ion Library TaqMan Quantitation Kit (Thermo Fisher Scientific). Following qPCR, the libraries were calculated and pooled together at equal 50 pM concentration for templating on the Ion Chef using the Ion 540 Kit-Chef (2 sequencing runs per initialization; Thermo Fisher Scientific). The samples were then sequenced on the Ion GeneStudio S5 System. Ion Reporter Software was used for mutation load and variant profiling analysis.

### IHC.

FFPE tissue samples were sectioned at a thickness of 5 μm, baked, and placed on positively charged glass slides. Slides were loaded on a Ventana DISCOVERY ULTRA (Ventana Medical Systems, Roche Diagnostics) automated IHC staining machine for deparaffinization, rehydration, endogenous peroxidase activity inhibition, and antigen retrieval (pH 8.5). Antigens were sequentially detected and heat inactivation was used to prevent antibody cross-reactivity between the same species. Following each primary antibody incubation (CD8, clone SP57; PD-L1, clone SP263; CD20, clone L26), DISCOVERY anti-Rabbit HQ or DISCOVERY anti-Mouse HQ and DISCOVERY anti-HQ-HRP were incubated. The stains were then visualized with DISCOVERY ChromoMap DAB Kit, DISCOVERY Teal Kit, and DISCOVERY Purple Kit, respectively; counterstained with hematoxylin (Ventana); and sealed with coverslips. Slides were imaged using the Vectra 3 automated quantitative pathology imaging system (Akoya Biosciences). Slides were then scored for percentage tumor stroma infiltration by a board-certified pathologist. In order to normalize percentage T_EX_ infiltration abundance for NanoString analysis in Figure 4, percentage T_EX_ of tumor tissues was calculated as follows: (FACS %T_EX_) × (IHC %CD8).

### Data availability.

All the single-cell RNA-sequencing data and NanoString data are uploaded in the GEO database. All R scripts used in this publication are available in https://github.com/weihuaguo/TEXinERpBC, commit ID 34a2bc30c9259e4cc7141e873cfc05802665caae.

### Statistics.

Graphs were created and statistics were performed using GraphPad Prism 8 and specific R packages as described. Statistics described were generated using 1-tailed unpaired Student’s *t* tests, Wilcoxon’s rank-sum tests, or 1-way ANOVAs with Holm-Šídák multiple-comparison *t* tests. Calculated *P* values are displayed as *, *P* < 0.05; **, *P* < 0.01; ***, *P* < 0.001; ****, *P* < 0.0001. A *P* value of less than 0.05 was considered significant. For all graphs, the mean is represented by a horizontal line. When shown, error bars represent ± SEM. When shown, the box plots depict the minimum and maximum values (whiskers), the upper and lower quartiles, and the median. The length of the box represents the interquartile range. Experiment-specific detailed statistical methods are described in corresponding figure legends and Methods sections.

### Study approval.

Fresh tumor and peripheral blood were obtained from patients who gave institutional review board–approved (IRB-approved) written informed consent prior to inclusion in the study (City of Hope IRB 05091, IRB 07047, and IRB 14346).

## Author contributions

CAE, WG, and PPL designed research studies and wrote the manuscript. CAE, WG, CA, DLS, JT, MHL, YJH, MSN, AC, and DBS conducted experiments; acquired data; and analyzed data. DBS, JHY, LK, LM, KM, JEM, YY, and JRW provided clinical sample acquisition support and translational feedback.

## Supplementary Material

Supplemental data

## Version 1 02/08/2022

Electronic publication
