# Supplementary material for: Tumor-infiltrating exhausted CD8+ T cells dictate reduced survival in premenopausal estrogen receptor–positive breast cancer
Source: JCI Insight. 2022 Feb 8;7(3):e153963. doi: 10.1172/jci.insight.153963 (PMC8855819; doi:10.1172/jci.insight.153963)
Supplement: Supplemental data [file jciinsight-7-153963-s091.pdf]

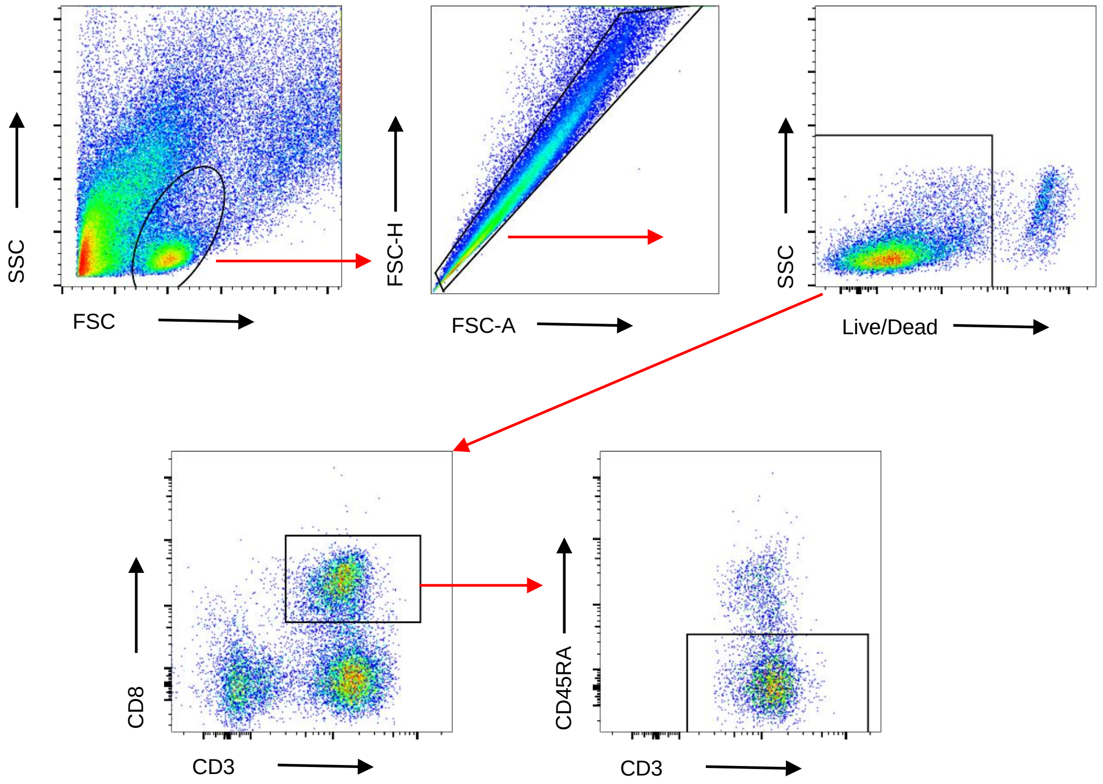

Supplemental Figure 1. Gating strategy for CD8+ T cells prior to analysis of PD-1 and CD39 expressing subsets.

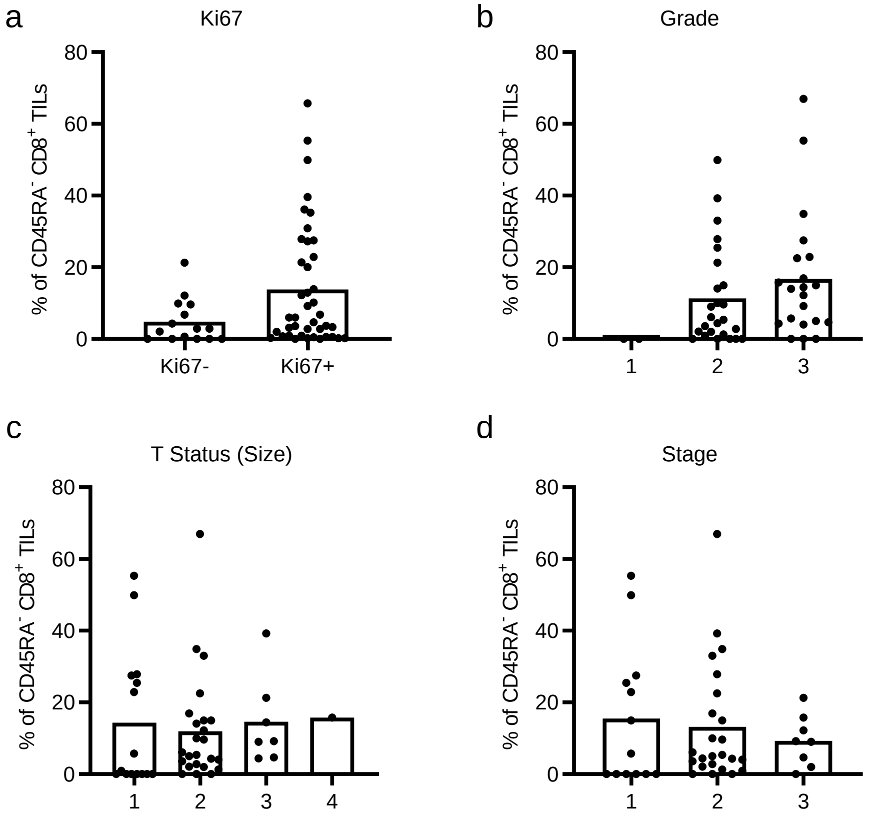

Supplemental Figure 2. Frequency of PD-1+ CD39+ cells within CD8+ TILs in ER+ tumors. Frequencies of memory (CD45RA-) CD8+ TILs in ER+ tumors are stratified by **a,** Ki67 scoring **b,** tumor grade **c,** T status (tumor size) and **d,** patient staging (n=66).

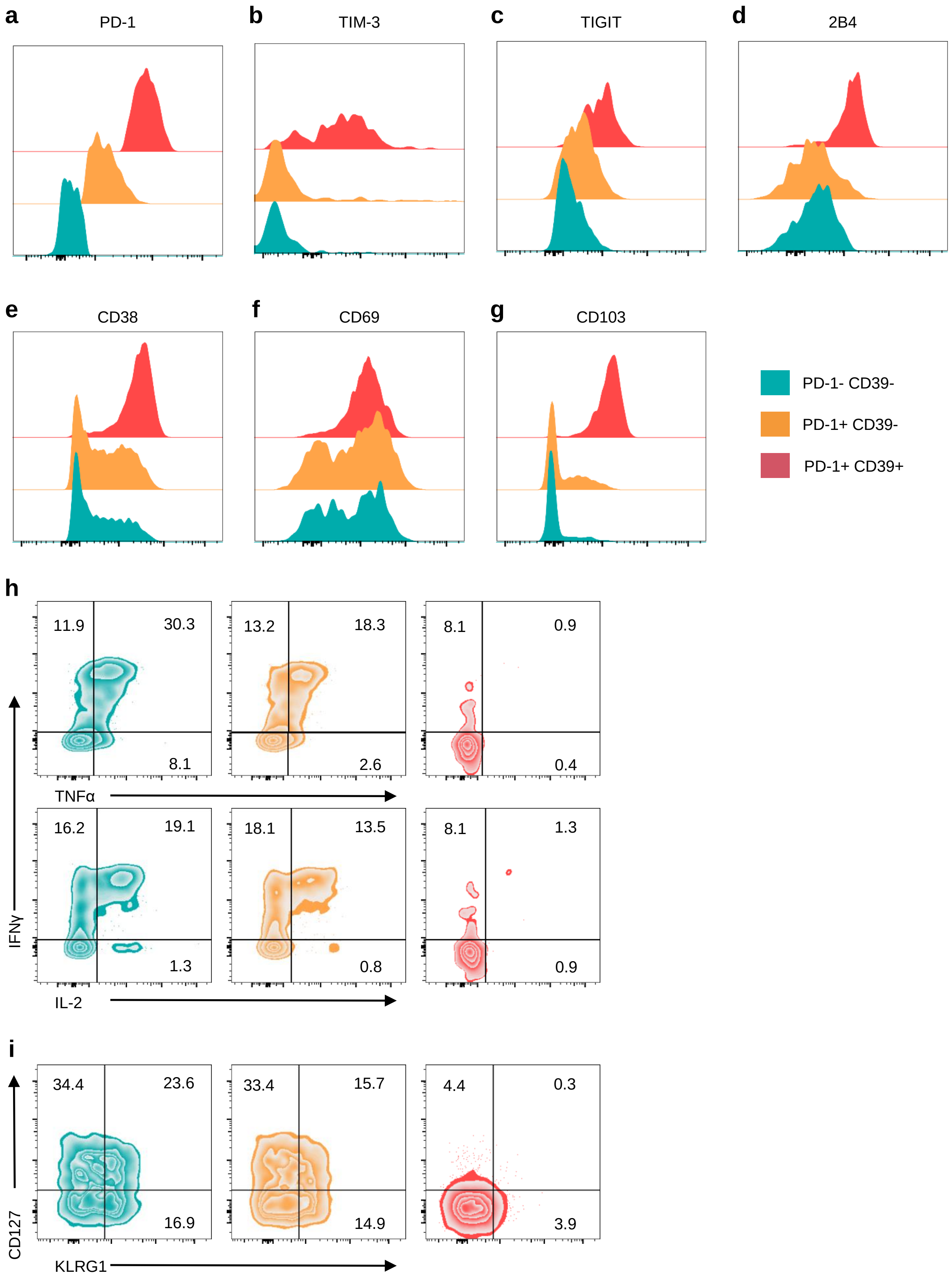

Supplemental Figure 3. Representative flow cytometry plots for protein expression of CD8+ TIL subsets. CD8+ TILs were gated for PD-1- CD39- (blue), PD-1+ CD39- (orange), and PD-1+ CD39+ (red) subsets. These subsets were then assessed for expression of **a,** PD-1, **b,** TIM-3 **c,** TIGIT **d,** 2B4 **e,** CD38 **f,** CD69, and **g,** CD103. CD8+ TILs were stimulated with PMA and ionomycin and assessed for production of **h,** IFNγ, TNFα, and IL-2 by intracellular flow cytometry. CD8+ TILs were also assessed for markers of T cell differentiation **i,** CD127 and KLRG1.

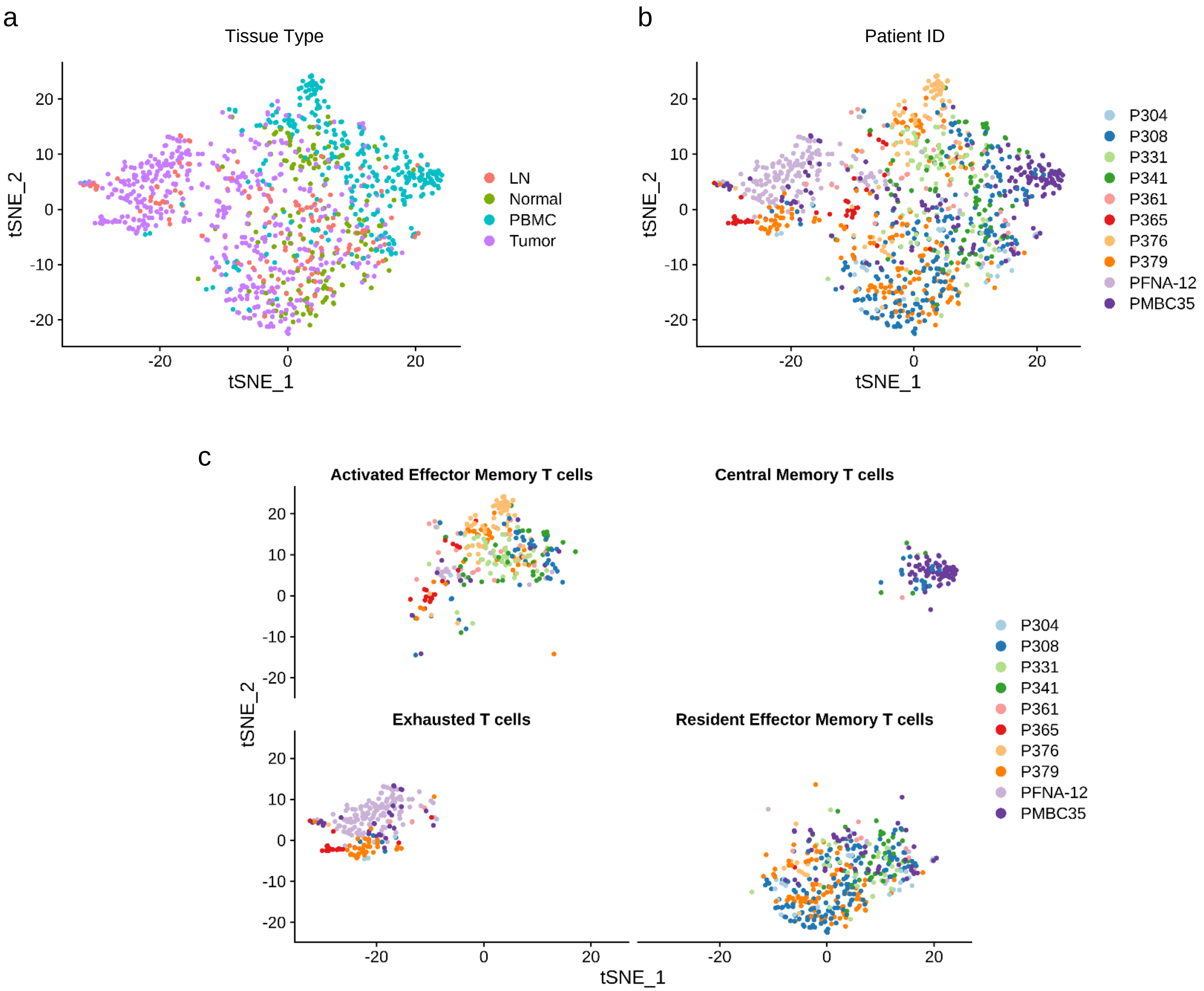

Supplemental Figure 4. Batch effect analysis of single cell cluster identification. tSNE projections of CD8+ T cells assayed by single cell sequencing are shown overlaid with **a,** tissue origin of cell **b,** patient ID and **c,** patient ID with each T cell subset overlaid individually. All patient tumors were ER+, except P379 and P365, which were TNBC.

a b

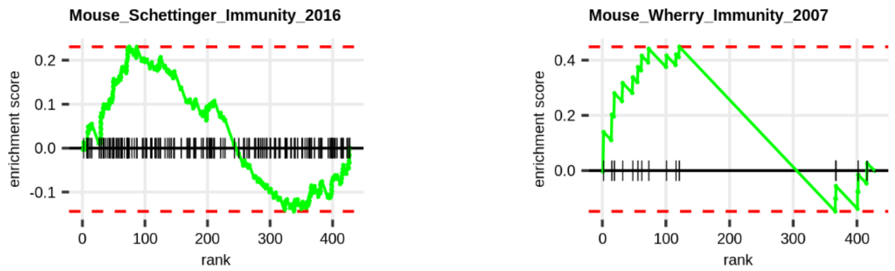

Supplemental Figure 5. Gene Set Enrichment Analysis of exhaustion signatures. Breast tumor Tex differentially expressed genes were compared to published gene expression data from two publications utilizing a LCMV murine model of chronic exhaustion: **a,** Schettinger, Immunity 2016 **b,** Wherry, Immunity 2007. Gene rank shown is from the current dataset.

a

		B-cells	CD8 T cells	Cytotoxic cells	DC	Exhausted CD8	Macrophages	Mast cells	Neutrophils	NK CD56dim	NK cells	T-cells	Th1 cells	Treg
T_{EX}	R	0.376	0.494	0.466	0.212	0.411	0.245	0.015	-0.056	0.507	0.372	0.422	0.523	0.475
	P	0.024	0.002	0.004	~~0.216~~	0.013	~~0.150~~	~~0.931~~	~~0.744~~	0.002	0.026	0.010	0.001	0.003
CD8	R	0.355	0.540	0.556	0.234	0.505	0.411	-0.052	-0.122	0.476	0.340	0.548	0.539	0.367
	P	0.034	0.001	0.000	~~0.170~~	0.002	0.013	~~0.763~~	~~0.480~~	0.003	0.042	0.001	0.001	0.028
PDL1	R	0.195	0.388	0.433	0.401	0.294	0.411	-0.074	-0.066	0.403	0.389	0.348	0.328	0.469
	P	~~0.255~~	0.019	0.008	0.015	~~0.081~~	0.013	~~0.668~~	~~0.704~~	0.015	0.019	0.037	~~0.051~~	0.004

b

		CCL5	*CD27*	*CD274*	*CD276*	*CD8A*	*CMKLR1*	*CXCL10*	*CXCL2*	*CXCL5*	*CXCL9*	*CXCR6*	*HLA-DQA1*	*HLA-E*	*IDO1*	*IRF4*	*LAG3*	*PDCD1LG2*	*PSMB10*	*STAT1*	*TIGIT*
T_{EX}	R	0.558	0.488	0.450	-0.376	0.496	0.167	0.515	-0.191	0.152	0.517	0.389	0.200	0.496	0.398	0.432	0.422	0.390	0.443	0.692	0.395
	P	~~<0.001~~	0.003	0.006	0.024	0.002	~~0.332~~	0.001	~~0.265~~	~~0.377~~	0.001	0.019	~~0.242~~	0.002	0.016	0.009	0.010	0.019	0.007	~~<0.0001~~	0.017
CD8	R	0.577	0.569	0.560	-0.298	0.570	0.290	0.559	-0.184	0.283	0.376	0.677	0.131	0.261	0.370	0.492	0.483	0.469	0.370	0.344	0.488
	P	<0.001	<0.001	~~<0.001~~	0.078	~~<0.001~~	~~0.086~~	<0.001	0.283	~~0.095~~	0.024	~~<0.0001~~	~~0.448~~	~~0.125~~	0.026	0.002	0.003	0.004	0.026	0.040	0.003
PDL1	R	0.387	0.364	0.436	-0.319	0.375	0.478	0.282	-0.278	0.220	0.288	0.421	0.029	0.439	0.342	0.323	0.394	0.496	0.357	0.305	0.414
	P	0.020	0.029	0.008	~~0.058~~	0.024	0.003	~~0.095~~	~~0.101~~	~~0.197~~	~~0.089~~	0.011	~~0.868~~	0.007	0.041	~~0.055~~	0.017	0.002	0.033	~~0.070~~	0.012

c

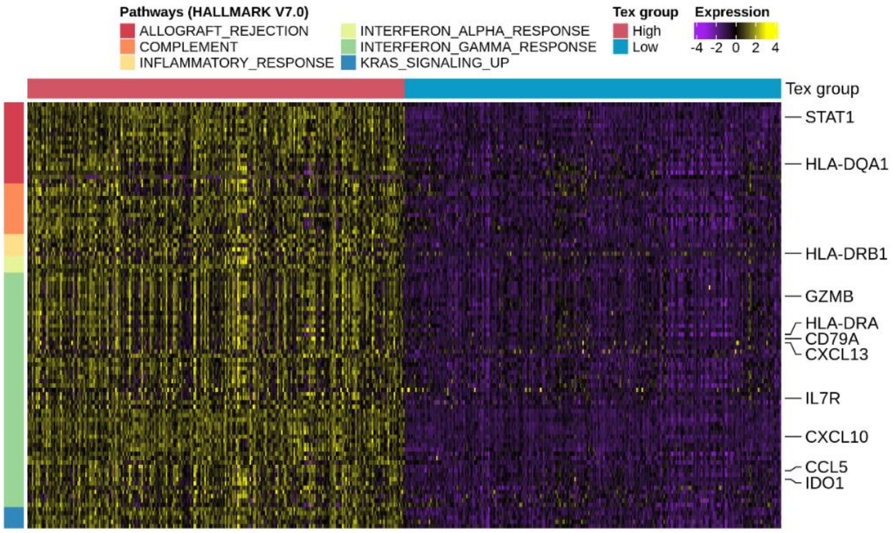

Supplemental Figure 6. Differential immune infiltration and gene expression in T_{EX}hi and T_{EX}lo tumors. ER+ breast tumors were assessed by Nanostring PanCancer Immune transcriptional profiling for correlations between **a,** absolute abundance of immune cell subsets or **b**, inflammation related genes and known abundance of CD8+ T_{EX}, CD8+ T cells, or PD-L1 expression (n=36). Correlation coefficients (R) values and p values are shown. P value text is strikethrough if p ≥ 0.05. **c,** METABRIC ER+ tumors were examined for differential gene expression between T_{EX} high (red; top 25%) and T_{EX} low (blue; bottom 25%). Hallmark pathways of differentially expressed genes (p<0.05) are annotated as described. Selected genes upregulated in T_{EX}hi tumors are called out.

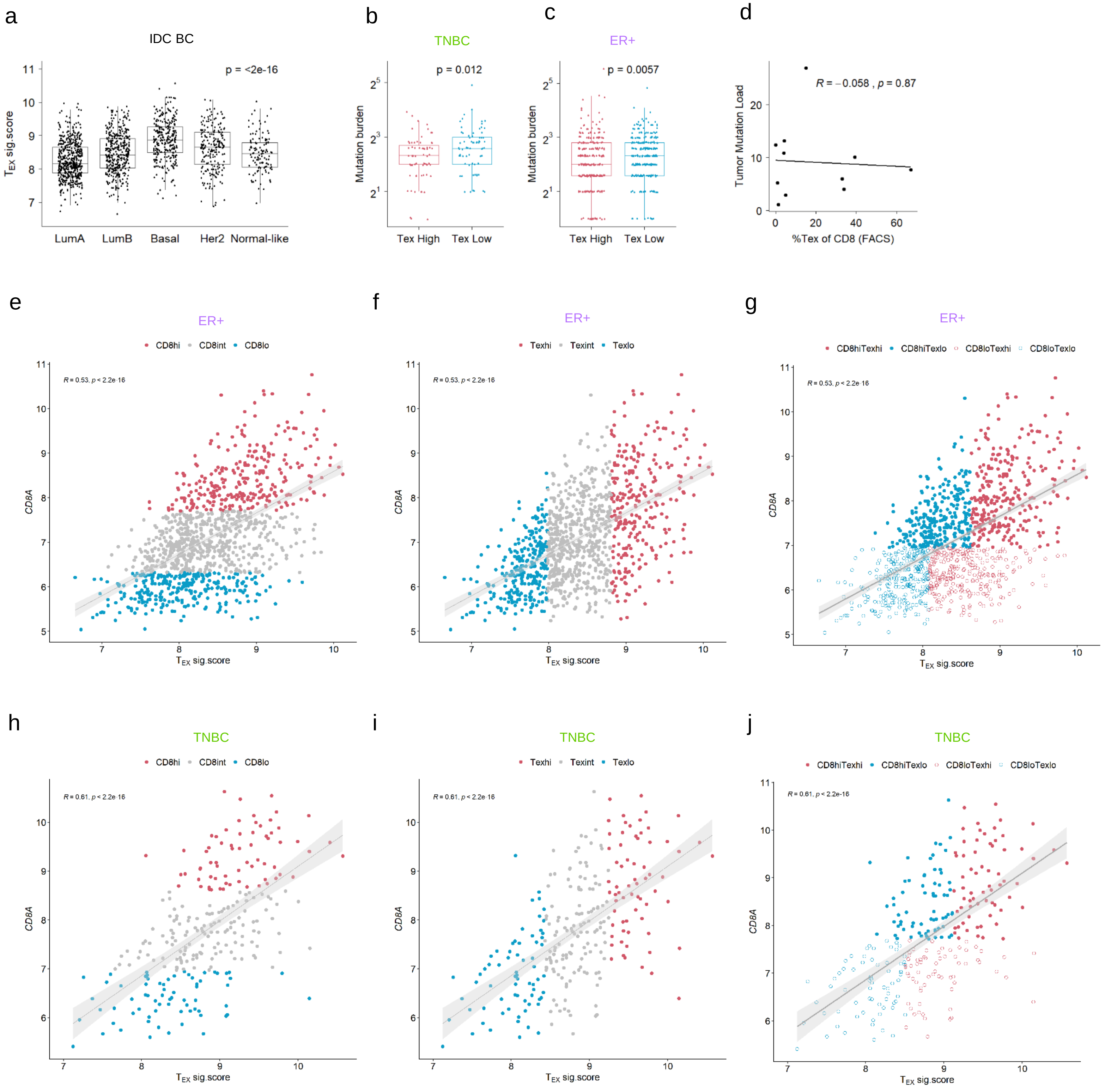

Supplemental Figure 7. Exhausted CD8+ T cell signature in breast cancer patients. a, CD8+ T cell exhaustion (T_{EX}) signatures were assessed for their prevalence in METABRIC cohort tissues in all invasive breast cancers by PAM50 molecular signatures of breast cancer. Mutation burden, or number of detected mutations detected, are shown for T_{EX} high (red; top 25%) and T_{EX} low (blue; bottom 25%) in **b,** TNBC and **c,** ER+ METABRIC breast tumors. **d,** Tumor mutation load (Oncomine software derived score) was also assessed in the context of %Tex of CD8+ TILs as assessed by flow cytometry in ER+ breast tumors. CD8 cutoff groups and TEX cutoff groups (top 25%, bottom 25%) from ER+ (**e,f**) and TNBC (**h,i**) METABRIC tumors are shown. Final four group composition of TEX hi and low (top 50%, bottom 50%) within CD8 hi and low (top 50%, bottom50%) are shown for ER+ (**g**) and TNBC (**j**) METABRIC tumors. Statistics were generated by one-way ANOVA (a,b), Wilcoxon Rank Sum test (c,d), Pearson's rank correlation (d), and non-parametric Spearman rank correlation (e-j).

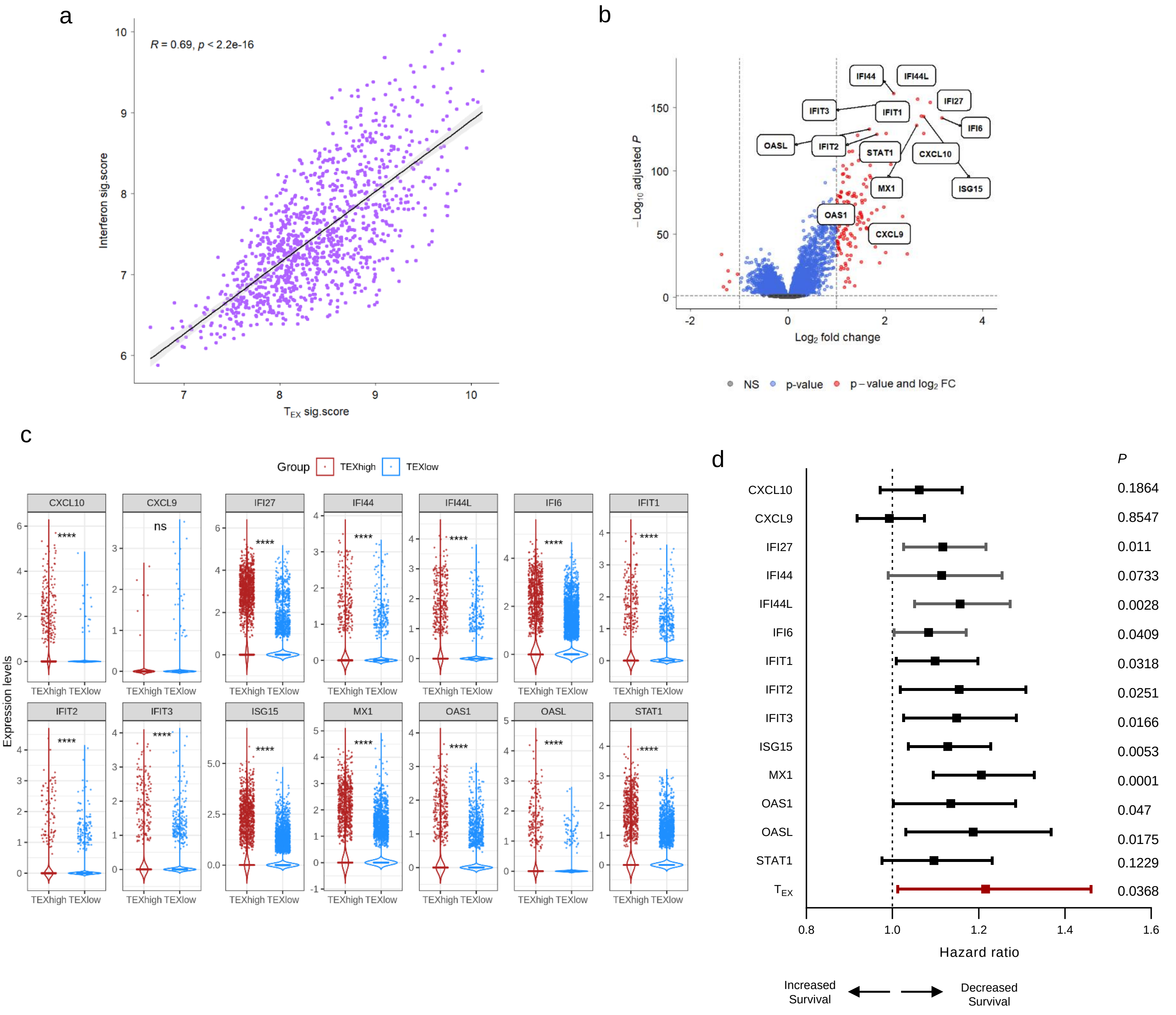

Supplemental Figure 8. Exhausted CD8+ T cell signature in breast cancer patients. a, METABRIC ER+ tumors were examined for correlation of of interferon gene expression signature and a T_{EX} gene signature expression. **b,** A volcano plot of differential gene expression with upregulated genes in T_{EX}hi tumors on the right, p-value cutoff of 0.01. Select interferon-γ related genes are called out. **c,** Expression levels of IFNγ related genes in cancer cells within T_{EX}hi tumors T_{EX}lo tumors, as determined by flow cytometry median, was assessed by single cell RNA sequencing. **d,** Cox hazard ratios for IFNγ related genes in regard to overall survival in METABRIC ER+ BC patients. Statistics generated with non-parametric Spearman rank correlation (**a**), Deseq (**b**), and Wilcoxon rank sum test (**c**).

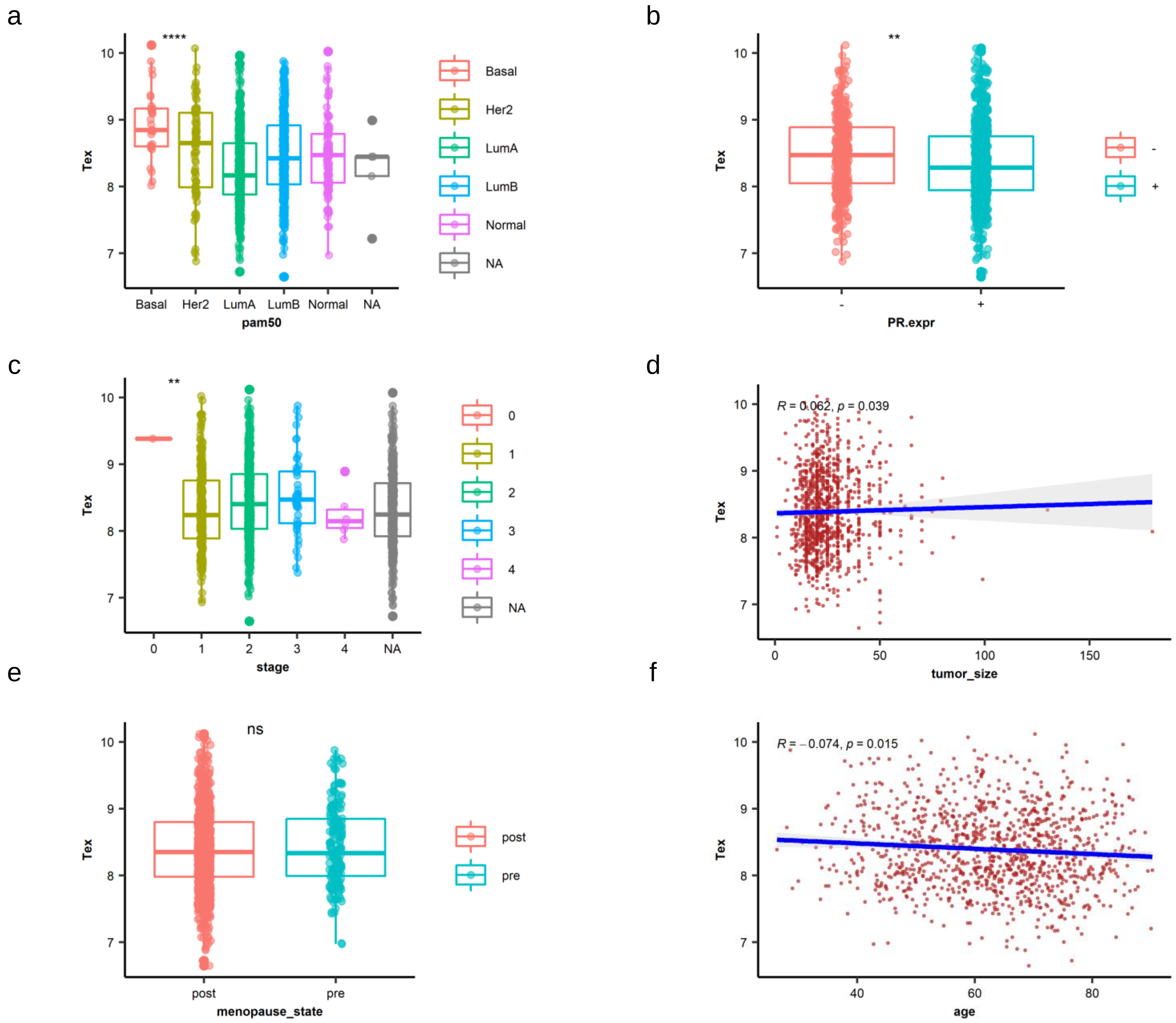

Supplemental Figure 9. Molecular and pathological features of ER+ BC tumors as they associate with infiltration of CD8+ T_{EX}. The T_{EX} gene signature expression level was assessed in METABRIC ER+ BC tumors within **a,** PAM50 molecular subsets **b,** tissue pathology progesterone receptor (PR) expression **c,** patient staging **d,** tumor size **e,** menopausal status and **f,** age. Statistics generated with one-way ANOVA (**a-c, e**) and non-parametric Spearman rank correlation (**d, f**).

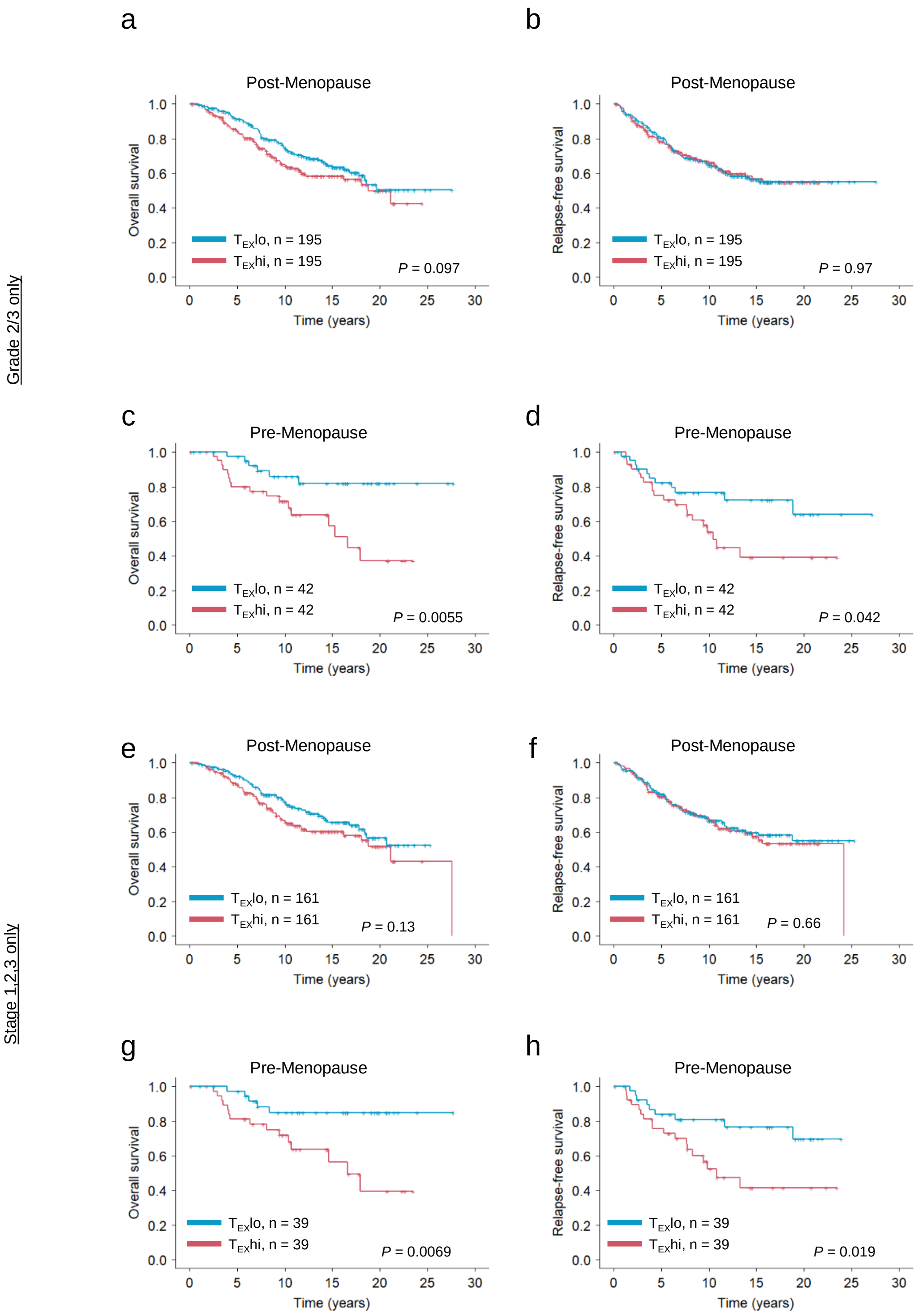

Supplemental Figure 10. Expanded survival characteristics of pre-menopausal and post-menopausal ER+ BC patients. METABRIC defined post-menopausal ER+ patient tumors were stratified by T_{EX} signature expression into T_{EX}hi (top 25%) and T_{EX}lo (bottom 25%) groups to examine overall survival and relapse-free survival in **a-b,** post-menopausal and **c-d,** pre-menopausal BC patients with Grade 2/3 only tumors. Overall survival and relapse-free survival in **e-f,** post-menopausal and **g-h,** pre-menopausal BC patients with Stage I-III only tumors.

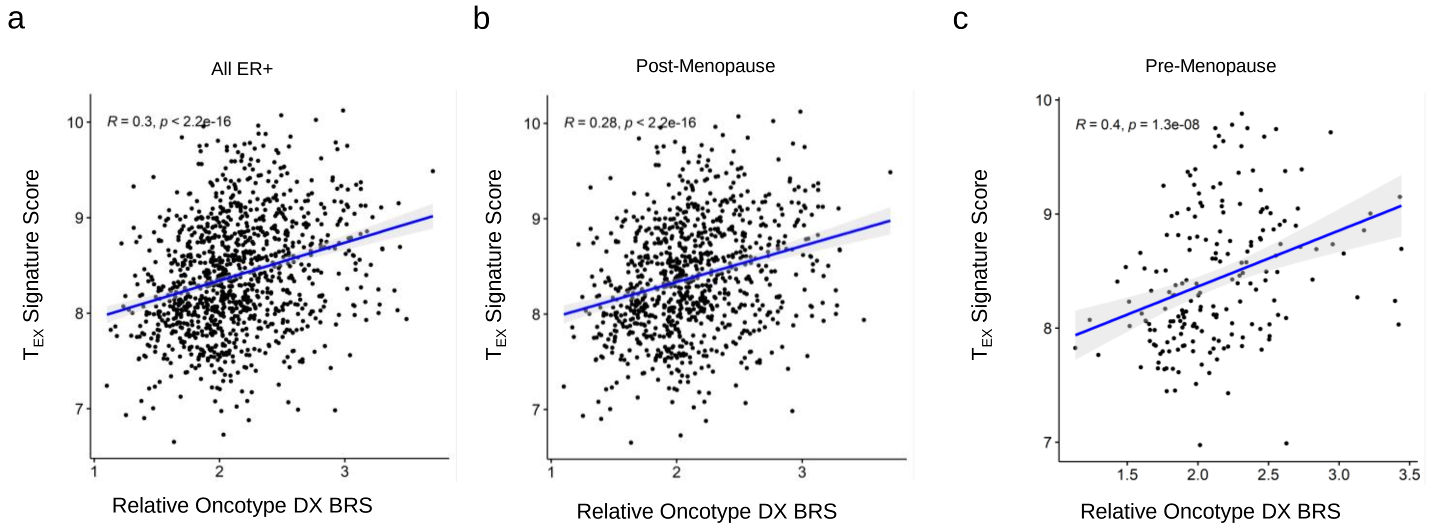

Supplemental Figure 11. Relationship between T_{EX} signature expression and Oncotype DX breast recurrence score in ER+ breast tumors. Relative Oncotype DX breast recurrence scores (BRS) and T_{EX} gene signature scores from METABRIC ER+ BC tumors within **a,** all ER+ patients **b,** post-menopausal ER+ patients and **c,** pre-menopausal ER+ patients. Statistics generated with Pearson's correlation.

a

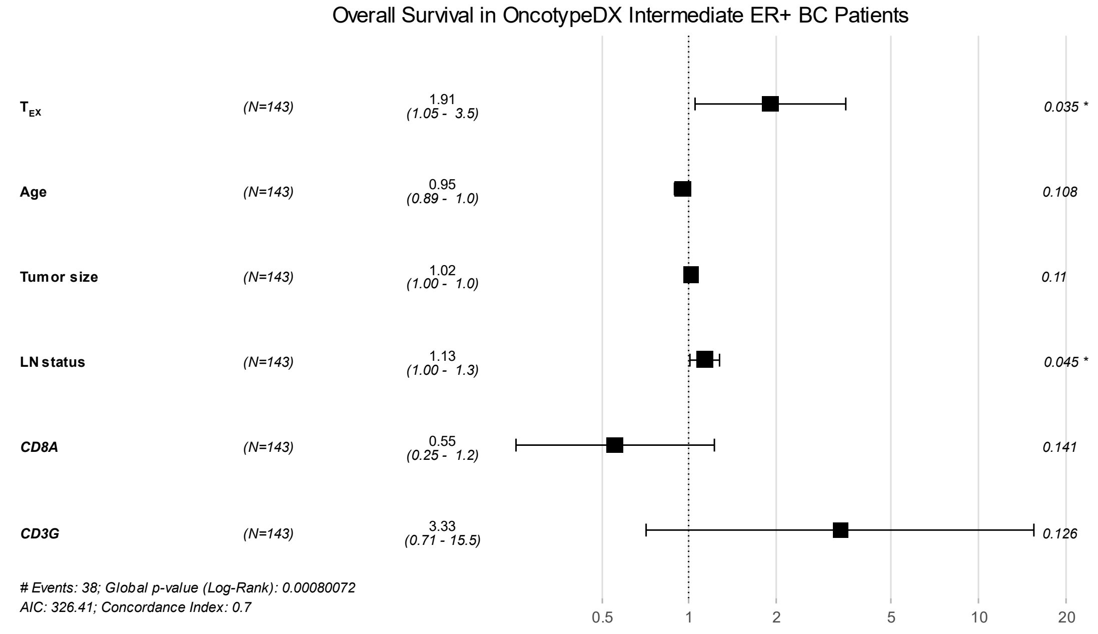

b

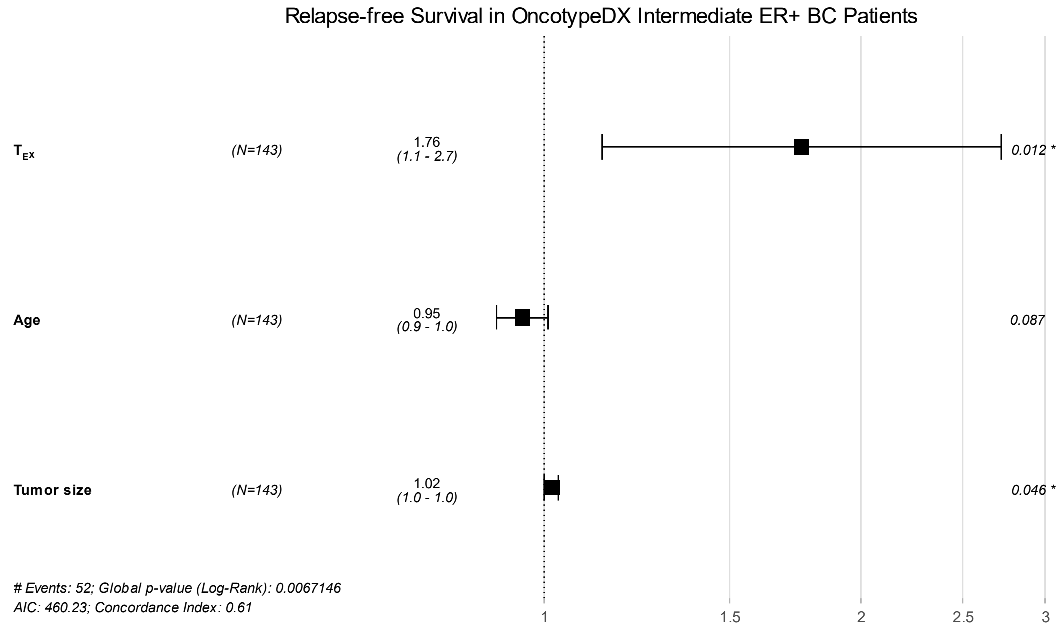

Supplemental Figure 12. Multivariate regression model to assess the relationship of T_{EX} and survival in ER+ breast cancer patients. A hybrid version of stepwise model selection was used to prune the model so that only necessary covariates were included. Akaike Information Criterion, or AIC, was used to select the best fitted model. Forest plots of estimated hazard ratios were generated for final fitted models. Schoenfeld residual global p-values was used to test for proportional hazard assumption. Results are shown for influence of variables on **a,** overall survival and **b,** relapse=free survival.

Tumor Tissue Breast Cancer Patient Characteristics		
**Age (years)**		
Mean, Median (Range)	54, 51 (29-86)	
**Pathological Subtype**		
ER+	n=70	
ER- PR- HER2- (TNBC)	n=12	
	ER+	TNBC
**Overall Stage**		
I	n=16	n=1
II	n=36	n=5
III	n=14	n=6
Unknown	n=2	n=0
**Pathologic Tumor Stage**		
T1	n=24	n=2
T2	n=35	n=7
T3	n=9	n=1
T4	n=1	n=2
Unknown	n=1	n=0
**Tumor Grade**		
1	n=3	n=0
2	n=36	n=1
3	n=29	n=11
Unknown	n=1	n=0

Supplemental Table 1. Clinical characteristics of breast cancer patient tumor samples for fresh tissue studies.

Flow Cytometry			
**Antibody**	**Clone**	**Fluorophore**	**Company**
PD-1	EH1.2	PE	BD Biosciences
CD39	A1	APC/FITC	Biolegend
TIM-3	F38-2E2	BV605	Biolegend
TIGIT	MBSA43	PerCPeF710	ThermoFischer
2B4	C1.7	PE-Cy7	Biolegend
CD160	BY55	AF488	BD Biosciences
CD69	FN50	BV605	Biolegend
CD103	Ber-ACT8	PE-Cy7	Biolegend
CD38	HB-7	FITC	Biolegend
CD127	A019D5	AF647	Biolegend
KLRG1	SA231A2	FITC	Biolegend
IFNγ	B27	AF700	BD Biosciences
TNFα	MAb11	BV421	Biolegend
IL-2	MQ1-17H12	FITC	BD Biosciences
CCR7	G043H7	BV421	Biolegend
CD3	BUV496	UCHT1	BD Biosciences
CD8	BUV805	SK1	BD Biosciences
CD45RA	HI100	APC-Cy7	Biolegend
Dead Cell Marker	Zombie	Zombie Red/ Zombie Blue	Biolegend

Supplemental Table 2. Reagents used for flow cytometry.

T_{EX} Gene Signature	
Gene	Log2 Fold Change
CXCL13	2.352554
GZMB	1.968331
IFI6	1.599293
HLA-DRA	1.34022
HLA-DQA2	1.284423
HLA-DRB5	1.417609
PRF1	1.37447
MX1	1.424808
HLA-DRB1	1.132757
CD82	1.268757
LY6E	1.217912
IFIT3	1.356663
ISG15	1.328407
IFI44	1.134001
IFITM3	1.322237
ENTPD1	1.17031
OAS1	1.162916
IFI44L	1.087082
BST2	1.16789
GNLY	1.301843
HAVCR2	1.262492
KRT86	1.48776
ALOX5AP	1.009562
CCL3	1.280291
IFI27	1.381946

Supplemental Table 3. 25 Gene Signature of CD8+ T_{EX}.

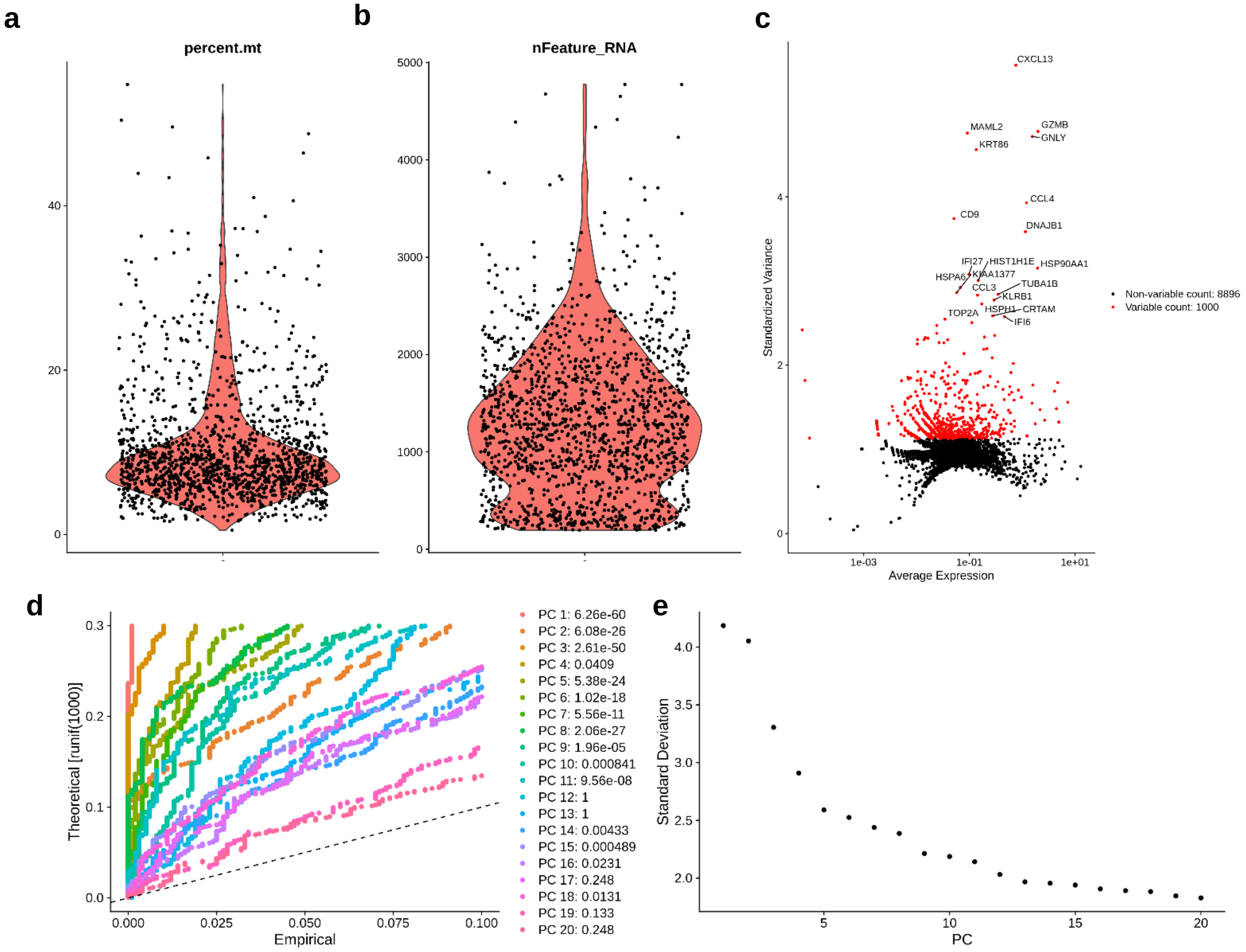

Supplemental Methods 1. Single-cell RNA-sequencing data quality control and principal component selection. Violin plot for number of non-zero expression genes for each cell, **a**. Violin plot for percentage of mitochondrial gene expression of each cell, **b**. Selected top 1,000 most variable genes (red dots) for PCA (top 20 are labeled with gene names), **c**. Modified Jack Straw method for PC selection, **d**. Elbow plot for PC selection (i.e., a ranking of principle components based on the percentage of variance explained by each one**)**, **e.**
